# Supplementary material for: 3DIANA: 3D Domain Interaction Analysis: A Toolbox for Quaternary Structure Modeling
Source: Biophys J. 2016 Jan 7;110(4):766–75. doi: 10.1016/j.bpj.2015.11.3519 (PMC4775853; doi:10.1016/j.bpj.2015.11.3519)
Supplement: Document S1. Supporting Materials and Methods and Figs. S1–S8 [file mmc1.pdf]

Biophysical Journal, Volume *110*

Supplemental Information

3DIANA: 3D Domain Interaction Analysis: A Toolbox for Quaternary Structure Modeling

Joan Segura, Ruben Sanchez-Garcia, Daniel Tabas-Madrid, Jesus Cuenca-Alba, Carlos Oscar S. Sorzano, and Jose Maria Carazo

Biophysical Journal

Supporting Material

3DIANA: 3D Domain Interaction Analysis: A Toolbox for Quaternary Structure Modeling

Joan Segura,[1,*] Ruben Sanchez-Garcia,[1] Daniel Tabas-Madrid,[1] Jesus. Cuenca-Alba,[1] Carlos Oscar S. Sorzano,[1] and Jose Maria Carazo[1]

[1]GN7, Spanish National Institute for Bioinformatics (INB) and Biocomputing Unit, National Center of Biotechnology (CSIC)/Instruct Image Processing Center, Madrid, Spain

S1 3DIANA web interfaces

This section presents the different graphical interfaces of the different tools integrated in 3DIANA web platform (see Section 2.2 in the main manuscript).

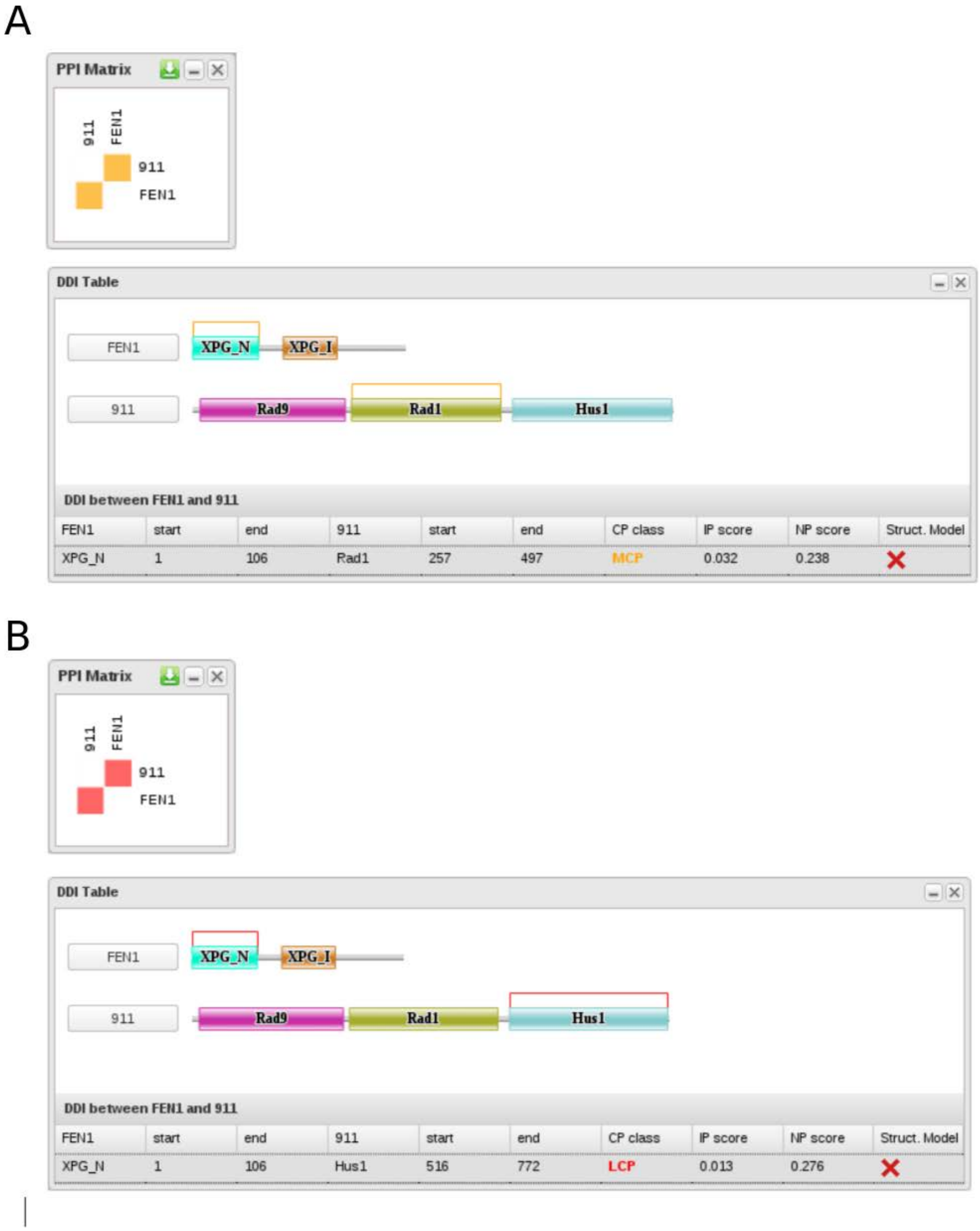

Figure S1: 3DIANA 'Domain-Domain Binding Analysis' tool. (A) Evaluation of the physical interactions between the subunits and domains in the proposed model (see Figure 2A main manuscript). The PPI matrix in the upper left corner displays the possible physical interactions between the different subunits, in this case 911 and FEN1, and the color indicates the best scored interaction between the domains of the two subunits (orange corresponds to medium confidence predictions –MCP-). Interactions between domains are displayed in a table with their corresponding DIMERO scores. In this case, the interaction is between the XPG_N domain of FEN1 and Rad1 of 911 and the interaction is scored as MCP. (B) Evaluation of a different proposed model for the 911/FEN1 complex (see Figure 2B in main text). In this case, the interaction is between the XPG_N domain of FEN1 and hus1 of 911, and the interaction is scored as low confidence prediction (LCP); note that this possible interaction between the two subunits is displayed in red in the PPI matrix.

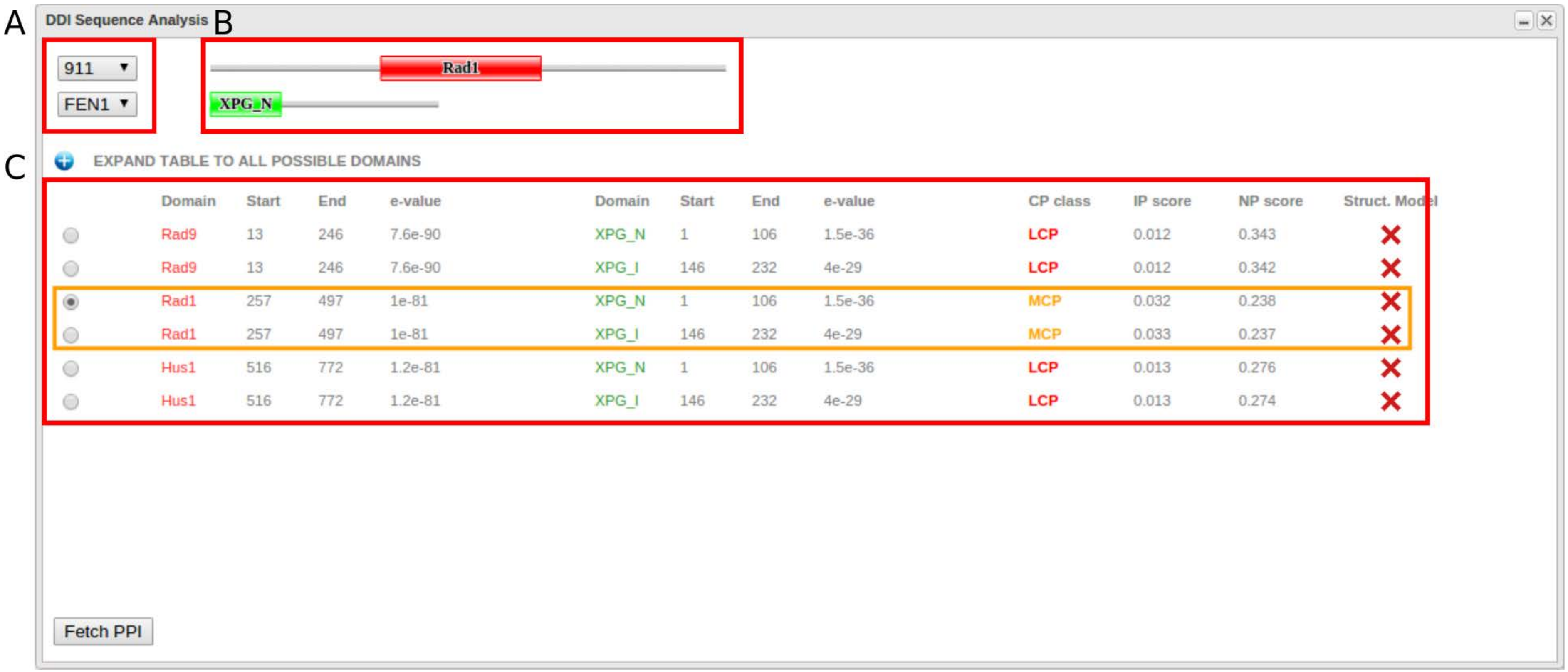

Figure S2: 3DIANA 'Domain-Domain Interacting Analysis' tool. 3DIANA GUI of the 'Domain-Domain Interacting Analysis' tool. (A) Selected pair of subunits in the analysis, in this example the 911 and the FEN1 subunits. (B) Graphical schema of the selected domain pairs (Rad1-XPG_N) in their respective sequences. (C) Domain-domain interaction table evaluating the probability of interaction between the domain pairs of the selected subunits (911 and FEN1); note that the best scored domain pairs (Rad1-XPG_N and Rad1-XPG_I) are shown within the orange rectangle)

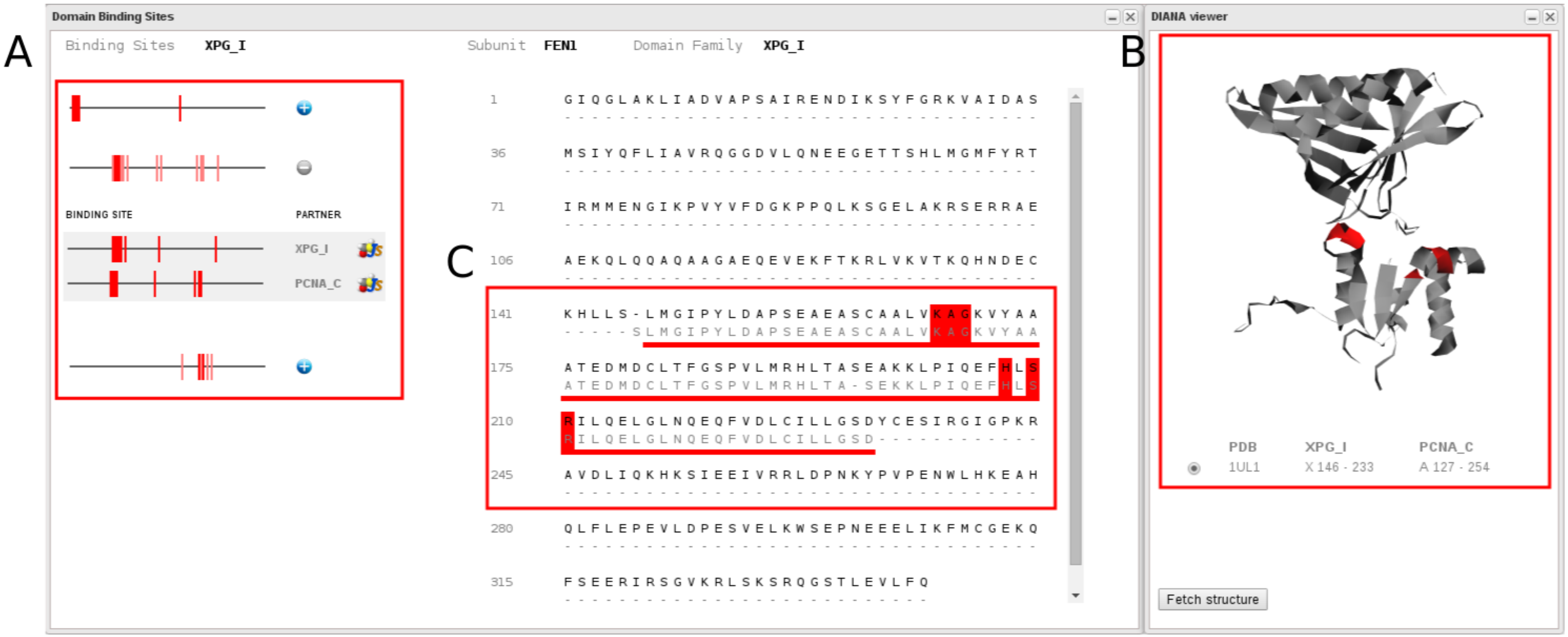

Figure S3: 3DIANA GUI of the 'Domain Binding Sites Analysis' tool. (A) Clustering of the different binding sites of the XPG_I domain family, the domain sequence is represented with a black line and the interacting residues of the binding sites are marked with perpendicular red segments. Each of the clusters is defined by different interactions between XPG_I domains and other domain partners. (B) Mapping of the potential interacting residues of XPG_I domain in FEN1 when XPG_I interacts with a PCNA domain, this information is derived from the DDI template contained in the PDB structure 1UL1; note that the alignment between both XPG_I (FEN1 and DDI template) domains is also displayed. (C) Structural viewer displaying the selected interactions, in this example the interaction between the XPG_I and PCNA domains contained in the PDB structure 1UL1.

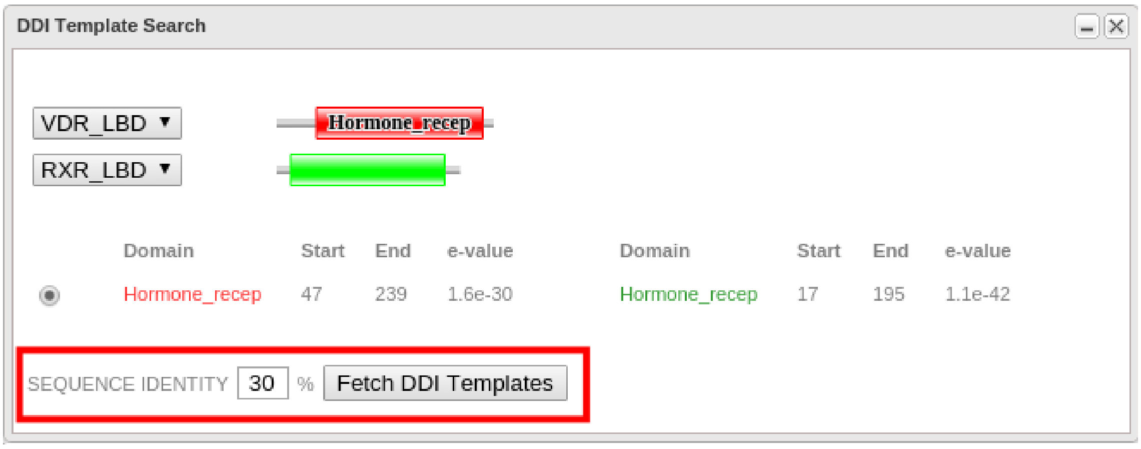

Figure S4: Domain-Domain Template Selection Interface. The interface allows to explore the available DDI templates for modeling the structure of the domains contained in the selected subunits. A threshold can be set up to filter those templates under a certain degree of sequence identity with the domains of interest.

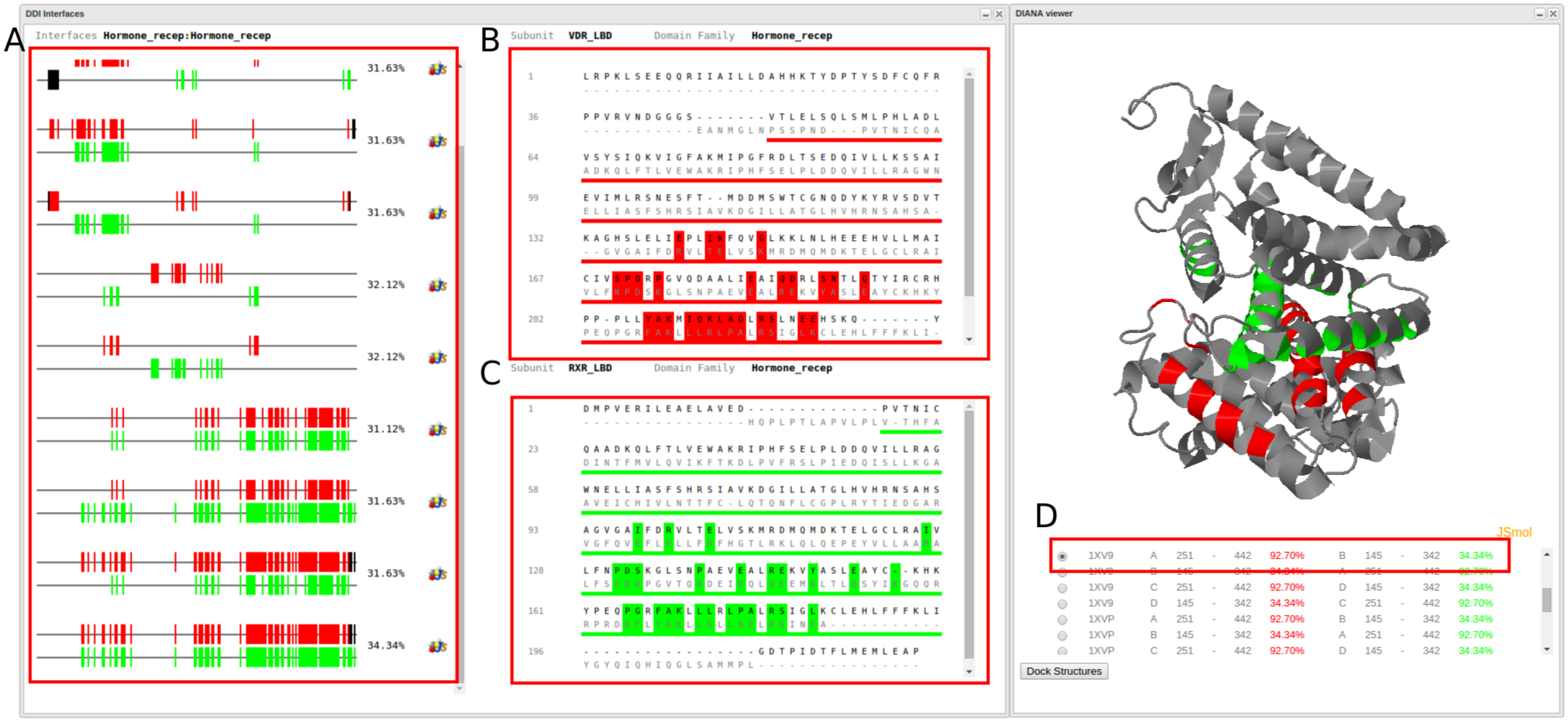

Figure S5: Domain-Domain Interaction Interfaces. (A) Templates are organized in clusters according to their interface conformation (see Section 2.1), also the highest sequence identity value of the templates compared with the domains of interest is displayed on the right. The interacting residues are represented in green and red segments along the domains sequence. (B,C) Sequence alignment of the selected template domains with the domains of interest underlined with a red and green lines. The interacting amino acids are highlighted in red and green colors. (D) 3D conformation of the selected DDI template, the interacting residues are mapped in red and green colors.

SM2 3DIANA examples using the 'Domain-Domain Binding Analysis' tool

In this section we present different cases of atomic structures solved by means of cryoEM and the corresponding results obtained with the 'Domain-Domain Binding Analysis' tool. The purpose of these analyses is to show how DIMERO predictions are in agreement with the reported conformations. However, it is worth to notice that an exhaustive benchmarking of DIMERO scores predicting DDIs was carried out in the work of Segura et al. (1) using atomic structures from the PDB (2).

The first analysis was performed on the VP16-Mediator-RNA polymerase II-TFII assembly in human (3)(EMDB code: EMD-5343). This structure (PDB code: 3J0K; see Figure S6A) is comprised by 12 distinct proteins and was used as an example of Scenario 1 (i.e., a hybrid model is proposed, and 3DIANA evaluates the likelihood of the proposed domain-domain interactions). From the proposed hybrid model, and in terms of physical binding between the protein domains, a total of 41 DDIs were observed. Out of these 41 domain pairs, 18 of them had a High Confidence Prediction level, and 23 had a Medium Confidence Prediction level. No proposed physical interaction was found to have a low likelihood. Therefore, this is the case of a proposed hybrid model that is very much in agreement with all previous interactomics knowledge as measured by DIMERO.

In the second example (Figure S7), the 'Domain-Domain Binding Analysis' tool was applied to the 40S-eIF1A-eIF1 complex from yeast (4) (EMDB code: EMD-3047). This structure (PDB code: 3JAM) is comprised by 36 different proteins and was also used as an example of Scenario 1. 3DIANA reported 49 interactions between the different protein domains where 46 of these domain pairs were classified as High Confidence Prediction (HCP), 2 of the binding domains as Medium Confidence Prediction and, finally, only one domain-domain interaction was classified as Not Significant (NS).

In these previous two examples we have shown how predictions of 3DIANA by means of DIMERO scores agrees with previously reported models. However, DIMERO is an statistical approach, and in some cases DIMERO scores may also lead to false predictions. To illustrate this case, we have analyzed the structure of the bacterial contractile nanotube present in *Pseudomonas aeruginosa* (5) (EMDB code: EMD-6270), showing the results in Figure S8. The atomic structure (PDB code: 3J9Q) is comprised of 48 chains represented by 2 unique proteins. The analysis found a total number of 234 interactions between the chains all of them involving a single DDI, thus the total number of DDI was also 234, but all of them were predicted as NS. The reason for this behavior is that only two different types of domain bindings were found: interactions between Phage_sheath_1 and Phage_tube domains and interactions between Phage_sheath_1 and Phage_sheath_1 domains. Both types of interactions are classified as NS using DIMERO and consequently all DDIs are classified as NS.

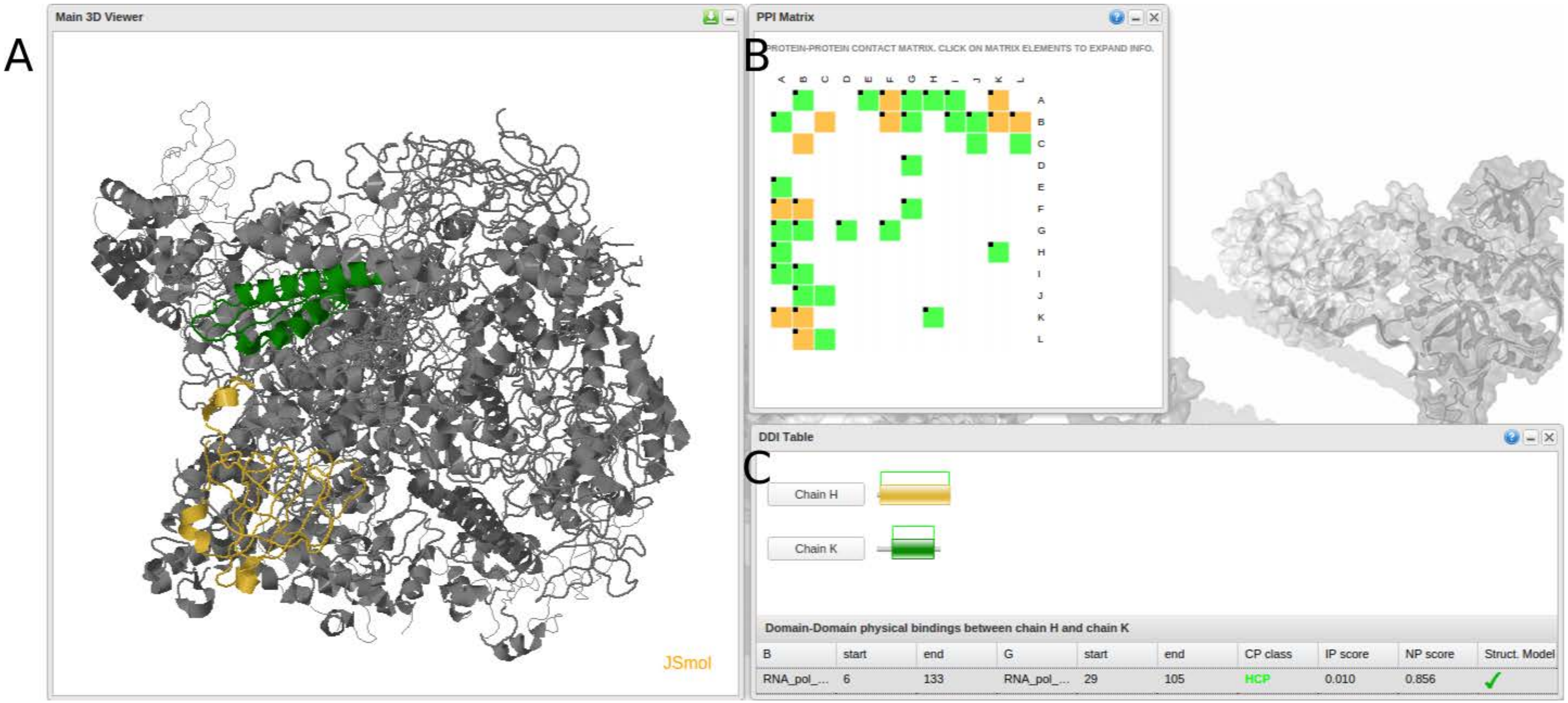

Figure S6: 3DIANA 'Domain-Domain Binding Analysis' results of the VP16-Mediator-RNA polymerase II-TFII assembly in human. (A) Atomic structure of the VP16-Mediator-RNA polymerase II-TFII complex (EMDB code: EMD-5343; PDB code: 3J0K). The 3D structure is displayed with the Jsmol viewer integrated in the 3DIANA web platform. (B) Protein-Protein Interaction Matrix displaying those interactions where at least a domain-domain binding occurs. The color code represents the best scored DDI using DIMERO approach (see Section 2.2 main manuscript) (C) Domain-Domain Interaction Table between chain H and chain K, only one DDI involving a RNA_pol_Rpb8 and RNA_pol_L_2 domain was observed between these chains. This domain pair was scored as HCP (see Section 2.2 main manuscript).

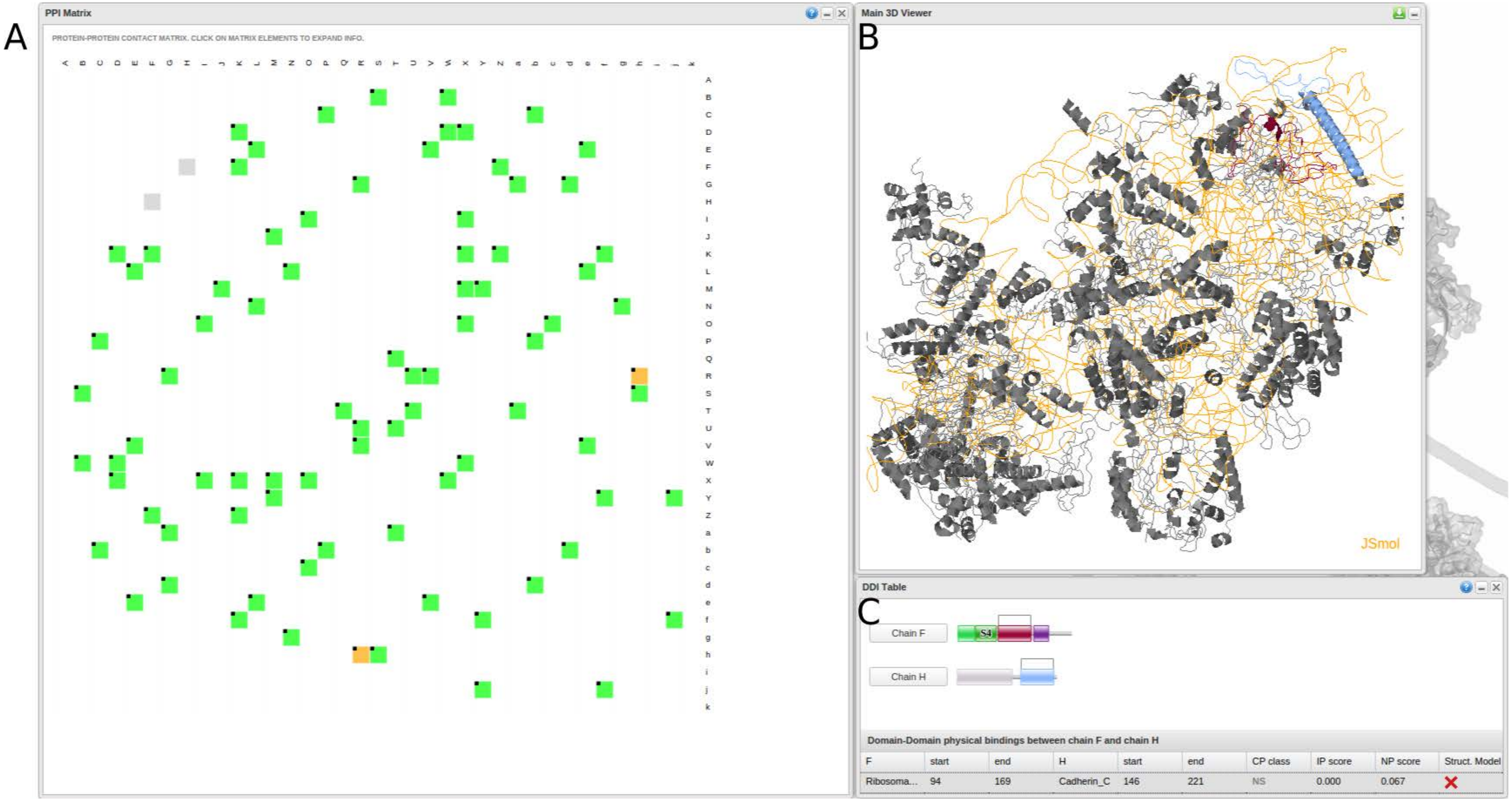

Figure S7: 3DIANA 'Domain-Domain Binding Analysis' results of the 40S-eIF1A-eIF1 complex in yeast. (A) Protein-Protein Interaction Matrix displaying those interactions where at least a domain-domain binding occurs. The color code represents the best scored DDI using DIMERO approach (see Section 2.2 main manuscript). (B) Atomic structure of the 40S-eIF1A-eIF1 complex (EMDB code: EMD-3047; PDB code: 3JAM). The 3D structure is displayed with the Jsmol viewer integrated in the 3DIANA web platform. (C) Domain-Domain Interaction Table between chain F and chain H, only one DDI involving a Ribosomal_S4e and Cadherin_C domain was observed between these chains. This domain pair was scored as NS (see Section 2.2 main manuscript).

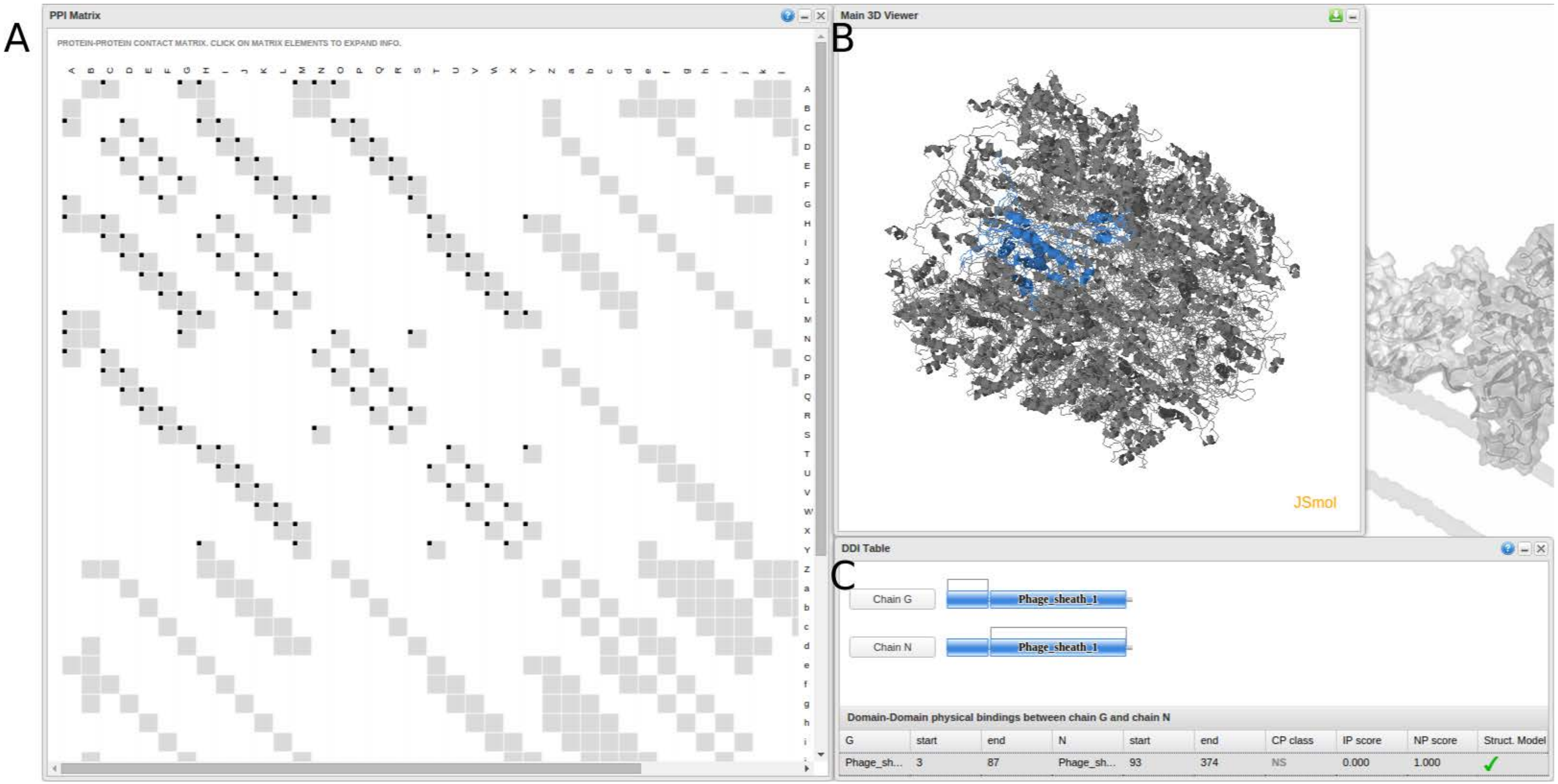

Figure S8: 3DIANA 'Domain-Domain Binding Analysis' results of bacterial contractile nanotube. (A) Protein-Protein Interaction Matrix displaying those interactions where at least a domain-domain binding is predicted to occur. The color code represents the best scored DDI using DIMERO (see Section 2.2 main manuscript). (B) Atomic structure of the bacterial contractile nanotube (EMDB code: EMD-6270; PDB code: 3J9Q). The 3D structure is displayed with the Jsmol viewer integrated in the 3DIANA web platform. (C) Domain-Domain Interaction Table between chain G and chain N, only one DDI involving two Phage_sheath_1 domains was observed between these chains. This domain pair was scored as NS (see Section 2.2 main manuscript).

References

1. Segura, J., C. O. Sorzano, J. Cuenca-Alba, P. Aloy, and J. M. Carazo. 2015. Using neighborhood cohesiveness to infer interactions between protein domains. Bioinformatics.
2. Gutmanas, A., Y. Alhroub, G. M. Battle, J. M. Berrisford, E. Bochet, M. J. Conroy, J. M. Dana, M. A. Fernandez Montecelo, G. van Ginkel, S. P. Gore, P. Haslam, R. Hatherley, P. M. Hendrickx, M. Hirshberg, I. Lagerstedt, S. Mir, A. Mukhopadhyay, T. J. Oldfield, A. Patwardhan, L. Rinaldi, G. Sahni, E. Sanz-Garcia, S. Sen, R. A. Slowley, S. Velankar, M. E. Wainwright, and G. J. Kleywegt. 2014. PDBe: Protein Data Bank in Europe. Nucleic acids research 42:D285-291.
3. Bernecky, C., P. Grob, C. C. Ebmeier, E. Nogales, and D. J. Taatjes. 2011. Molecular architecture of the human Mediator-RNA polymerase II-TFIIF assembly. PLoS Biol 9:e1000603.
4. Llacer, J. L., T. Hussain, L. Marler, C. E. Aitken, A. Thakur, J. R. Lorsch, A. G. Hinnebusch, and V. Ramakrishnan. 2015. Conformational Differences between Open and Closed States of the Eukaryotic Translation Initiation Complex. Mol Cell 59:399-412.
5. Lin, D. Y., J. Diao, and J. Chen. 2012. Crystal structures of two bacterial HECT-like E3 ligases in complex with a human E2 reveal atomic details of pathogen-host interactions. Proceedings of the National Academy of Sciences of the United States of America 109:1925-1930.
