# Supplementary material for: 3DIANA: 3D Domain Interaction Analysis: A Toolbox for Quaternary Structure Modeling
Source: Biophys J. 2016 Jan 7;110(4):766–75. doi: 10.1016/j.bpj.2015.11.3519 (PMC4775853; doi:10.1016/j.bpj.2015.11.3519)
Supplement: Document S2. Article plus Supporting Material [file mmc2.pdf]

Biophysical Journal Volume 110 February 2016 766–775

Article

3DIANA: 3D Domain Interaction Analysis: A Toolbox for Quaternary Structure Modeling

Joan Segura,[1,*] Ruben Sanchez-Garcia,[1] Daniel Tabas-Madrid,[1] Jesus Cuenca-Alba,[1] Carlos Oscar S. Sorzano,[1] and Jose Maria Carazo[1]

[1]GN7, Spanish National Institute for Bioinformatics (INB) and Biocomputing Unit, National Center of Biotechnology (CSIC)/Instruct Image Processing Center, Madrid, Spain

ABSTRACT Electron microscopy (EM) is experiencing a revolution with the advent of a new generation of Direct Electron Detectors, enabling a broad range of large and flexible structures to be resolved well below 1 nm resolution. Although EM techniques are evolving to the point of directly obtaining structural data at near-atomic resolution, for many molecules the attainable resolution might not be enough to propose high-resolution structural models. However, accessing information on atomic coordinates is a necessary step toward a deeper understanding of the molecular mechanisms that allow proteins to perform specific tasks. For that reason, methods for the integration of EM three-dimensional maps with x-ray and NMR structural data are being developed, a modeling task that is normally referred to as fitting, resulting in the so called hybrid models. In this work, we present a novel application—3DIANA—specially targeted to those cases in which the EM map resolution is medium or low and additional experimental structural information is scarce or even lacking. In this way, 3DIANA statistically evaluates proposed/potential contacts between protein domains, presents a complete catalog of both structurally resolved and predicted interacting regions involving these domains and, finally, suggests structural templates to model the interaction between them. The evaluation of the proposed interactions is computed with DIMERO, a new method that scores physical binding sites based on the topology of protein interaction networks, which has recently shown the capability to increase by 200% the number of domain-domain interactions predicted in interactomes as compared to previous approaches. The new application displays the information at a sequence and structural level and is accessible through a web browser or as a Chimera plugin at http://3diana.cnb.csic.es.

INTRODUCTION

Electron microscopy (EM) is experiencing a revolution with the advent of a new generation of Direct Electron Detectors (1) and the improvement of image processing algorithms, enabling a broad range of large and flexible structures to be resolved well below 1 nm resolution (2–4). Although EM approaches are evolving to a near-atomic resolution, for many macromolecular complexes the attainable resolution might not be enough to directly determine their structure at atomic level. The latter may be the case of either small or very flexible complexes studied by single particle analysis methods, or structures coming from subtomogram averaging. These medium resolution cases, above 5 Å resolution, are the target of this work. Still, in all cases, the link between EM maps and defined chemical entities is a necessary step toward a deeper understanding of the molecular mechanisms that allow proteins to perform specific tasks. For this reason, in those cases in which EM data do not lead themselves to near-atomic resolution structures, an effort is made to generate pseudoatomic models combining EM maps with high-resolution structures obtained with other techniques, generating the so called hybrid models. Many methods for the integration of medium resolution EM three-dimensional (3D) maps with high-resolution structures have been proposed, including determination of secondary structure elements and sequence assignment (5,6), modeling of missing backbone segments with experimental data (7,8) and fitting of x-ray or NMR structures into EM density maps (9–11). The case of EM maps with a resolution better than ~5 Å may be approached in a rather direct manner, in many cases leading to atomic-accuracy models (12,13). In this work the term fitting will be used to appoint the process of allocating atomic models into a density map, whereas the term docking will be used to describe the structural modeling of protein interactions.

Many medium resolution EM fitting approaches perform a six-dimensional search to localize the positions where the atomic structures maximize a certain scoring function with respect to the density map. The cross correlation between the atomic density and the EM map has been shown to be a good prediction score (14). However, other functions, including difference least squares, envelope scoring, or Laplacian-filtered correlation can be useful at different resolution levels (15). Many times, EM fitting involves multiple subunits and thus, protein-protein contacts need to be

Submitted July 1, 2015, and accepted for publication November 30, 2015.

*Correspondence: jsegura@cnb.csic.es

This is an open access article under the CC BY license (http://creativecommons.org/licenses/by/4.0/).

Editor: Edward Egelman.

© 2016 The Authors

0006-3495/16/02/0766/10

http://dx.doi.org/10.1016/j.bpj.2015.11.3519

considered to optimize the atomic interactions among the fitted structures. To satisfy biophysical restraints between the atomic contacts of the proteins, EM fitting is combined with protein docking methods. For example, ATTRACT-EM (16) combines a Gaussian overlap model to fit the subunits within an EM map with the atomic force field of the docking method ATTRACT (17) to refine the resulting poses. Another example is Multi-Fit (18). This latter approach consists of two steps. First, a segmentation of the density map so that the cross correlation between the segmented regions and the atomic structures is computed to determine their positions within the volume. Second, the docking method PatchDock (19), based on geometric complementarity, is used to refine the atomic contacts between the different proteins. Furthermore, EMfit (20) is a method that can perform global and local fitting by means of the sum of densities at atomic sites, the lack of atoms in low density regions, and the consideration of atomic clashes. Finally, some general docking methods can also include EM maps as additional restraints, improving the conformational search of solutions and adding additional terms to their scoring functions. A good example of the latter is the HADDOCK-EM (21) protocol, which incorporates a local cross correlation term to the energy-like scoring function of HADDOCK (22), a high accurate docking approach based on energy minimization and geometric restraints. A review of the results obtained during the first Modeling Challenge can be found in (23). It is in this context that we place our work, developing, to our knowledge, a novel approach to guide the fitting of high-resolution structures into medium resolution EM maps.

The novelty of the work we present is that we do not aim at providing a modeling method per se but, instead, we bring the knowledge on reported protein-protein-interactions (PPIs) to the fitting process, somehow mimicking the role of an expert in the complex under investigation who has at his fingerprints all the relevant published information. The current amount of interactomics data have been proved to be efficient to predict interactions between protein domains (24) and thus, useful to find potential contacts between interacting proteins. In addition, the number of experimentally resolved domain-domain interacting (DDI) structures covers a broad range of protein interactions (25) allowing to model their quaternary structure. The low computational cost of these statistical approaches allows a fast preliminary prediction of potential contacts between protein subunits or modeling the structure of interactions in a protein complex. However, in many cases statistical methods are not suitable when knowledge-based information is not available and thus, ab initio approaches based on structural geometry or biophysical models are needed. Furthermore, both types of approaches are not exclusive and can be combined to achieve more accurate results.

In this way, we present, to our knowledge, a new web platform integrating knowledge-based tools and prediction methods, such as interactomics data providing binding sites, potential interacting partners, and structural templates for template-based docking. This type of information may be helpful to discern among a number of different possible interactions between the different subunits of a protein complex, assisting to decide on their mutual orientations within the EM 3D map before starting a more detailed analysis with modeling and refinements tools (12,26). Moreover, the application includes a collection of structural templates between protein domains that can be used to model the structure of those proposed interactions between subunits involving domain family pairs with solved structures of their interaction. Finally, the web platform also integrates a fast and accurate protein-guided docking method, PatchDock (19), to provide a modeling tool when knowledge-based data is not available. The application is accessible through a user-friendly web interface at http://3diana.cnb.csic.es and, also, through Chimera viewer (27), installing a plugin developed to interface the web browser with the desktop viewer. We present in the main text two use cases that illustrate how the information provided by 3DIANA can be used to propose a first model of the quaternary structure of protein complexes (other examples are presented in the Supporting Material).

MATERIALS AND METHODS

3DIANA is a web platform that comprises a collection of knowledge-based and computational tools for the study of protein interactions and quaternary structure of protein complexes. Considering the limited resolution of the targeted EM maps, we mainly focus our work at the protein domain level rather than at individual amino acids. In this way, 3DIANA start point is the evaluation and analysis of all possible DDIs corresponding to the complex under study, calculated following a probabilistic approach where a large set of interactomics information has been taken into account (24). In this way, the user can easily recognize which DDIs can be mapped to solved structures or which is the most probable set of DDIs for a particular specimen at hand, finding starting points for building a first model or judging if a proposed structure is compatible, or not compatible, with the published body of biomedical information. Once a given domain-domain architecture has been selected, 3DIANA allows the user to explore instances in which experimental 3D structures corresponding to protein domains of the same type (of the same Pfam family) have been reported, so that these structures can be overlaid on the cryo-EM map, suggesting possible interacting templates. In this way, given one or more protein structures, the application allows to map known binding regions of their domains, perform structural docking based on DDI templates (28) and, finally, infer interactions among the different domain pairs of the proteins.

Building 3DIANA: external databases and methods

3DIANA is a web platform designed to provide experimental based as well as predicted interactomics data to assist users during the fitting of protein structures within an EM density map. The main utilities of the platform include the identification of those protein domains more likely to interact, the use of experimentally solved structures to model interactions between subunits, and resorting to a protein docking tool to predict

potential poses between proteins when no further experimental data is available. The information provided by the platform is related to protein domains and, thus, the first step is to determine the domains of the different subunits. Once the protein domains have been defined, 3DIANA evaluates which DDIs are more likely to occur using DIMERO scores (see *DIANA toolbox: protein interaction analysis tools*). Also, the 3DID (29) database has been integrated into the platform to check if any of the potential DDIs were experimentally solved and, in this case, to allow the use of these structures as templates to perform template-based docking with PatchDock (19).

3DIANA uses protein domains as a reference system to annotate experimental information and to infer new interactomics data. Multiple methodologies have been proposed to determine protein domains based on structural information (30,31) or protein sequences (32,33). In this work, protein domains are defined according to the Pfam classification (34), where domains are identified by sequence patterns using hidden Markov models (HMM). To delineate domains in protein sequences, 3DIANA includes the HMMER (35) package and the HMMs database of Pfam. Thereby, the first operation that 3DIANA performs when a complex formed by a defined set of proteins is submitted, is the computation of the whole set of protein domains.

Several studies have found that PPIs are mediated by a limited catalog of DDIs (36–43) and thus, DDI information can be useful to model interactions. However, the number of experimentally determined DDIs is limited (24,36), and for most PPIs there is no information about their possible interacting domains. To alleviate this problem, recently we have proposed DIMERO, a new approach to predict interactions between protein domains based on PPI networks and neighborhood cohesiveness. DIMERO is able to increase by 200% the fraction of DDI predictions available so far (24), providing higher reliability than previous methods. 3DIANA includes this approach to increase the range of experimental information, adding DIMERO predictions and providing an alternative source when no experimental data is available. In this manner, the user can predict potential contacts among the domains of different proteins when there is no structural evidence. Naturally, our approach, based on protein domains, cannot be directly applied to intrinsically disordered Proteins, because this is one of the limitations of this method.

DIMERO scores were calculated using STRING interactomics networks (44). The STRING database contains PPIs from experimental sources and prediction methods and currently it is one of the most comprehensive databases covering interactomics networks. This information has been integrated into 3DIANA, allowing the user to retrieve lists of PPI data involving two particular domains. Browsing these PPIs offers the possibility to find additional information about known interactions involving the two domains of interest, including experimental data, information from prediction methods, and scientific literature.

Once possible protein domain interactions have been identified, 3DIANA provides (annotate) additional information, such as interactomics data related to binding sites or directly proposes structural templates—if they exist—for protein docking. To that end, we have used the 3DID database (29) as a source of interactomics knowledge to annotate binding regions and to provide a collection of DDI templates for template-based docking. The 3DID database is a compilation of interacting domain pairs for which 3D coordinates are available from the Protein Data Bank (PDB) (45). 3DID includes intra- and intermolecular interactions between protein domains. However, the main purpose of 3DIANA is the study of interactions between proteins and therefore, only intermolecular interacting domain pairs were considered.

Homologous pairs of proteins often interact using similar interacting areas; however, they are not unique (46). To encapsulate this information, 3DID classifies DDI structures between two domain families in clusters according to the geometry of the DDIs (47). Domains within a Pfam family can then be aligned to its consensus sequence (most probable domain sequence in the family) and the interacting residues of the different domains can be mapped on the same reference. Finally, for each domain family, all clusters resulting from the interactions with the rest of the Pfam families are grouped again based on the number of common interacting residues mapped on the consensus sequence. As a result, each domain family comprises a collection of global binding sites where the interacting residues can be displayed on the consensus sequence.

3DIANA toolbox: protein interaction analysis tools

DDI analysis

This tool allows the evaluation of potential contacts/interactions between the domains of two selected proteins, highlighting which are the most probable domain pairs to interact and, therefore, to be used in more detailed modeling strategies. The application evaluates all possible domain pairs between the selected proteins independently of their structural conformation and physical contacts. The evaluation process is performed with DIMERO (24), a method that evaluates potential DDIs based on interactomics networks and neighborhood cohesiveness. DIMERO classifies DDIs in four different categories: high confident predictions (HCP), medium confident predictions (MCP), low confident prediction (LCP), and not significant (NS) scores. These scores were obtained by analyzing the statistical performance of the methodology in a particular benchmark (24) designed to evaluate the discriminative power to distinguish among interacting and noninteracting domain pairs. The different classification scores were chosen so that a domain pair classified as HCP, MCP, LCP, and NS had a probability lower than 5%, 10%, 15%, and 25%, respectively, to be a noninteracting domain pair. The tool displays all possible domain pair combinations with their position in the protein sequences and the computed DIMERO scores. Furthermore, when structural evidence exists for a particular domain pair, this is indicated in the table. Selecting the different elements of the table, the user can highlight concrete domain pairs in the protein structures. Finally, this tool can be used to browse the PPIs involving a particular domain pair, and directly accessing the STRING website to find additional information.

Domain-domain binding analysis

3DIANA can be used to analyze the likelihood of the proposed physical bindings occurring between the proteins and domains of a given candidate hybrid model. Interactions are calculated measuring the distances between nonhydrogen atoms, so that proteins or domains with any pair of nonhydrogen atoms closer than 6 Å are considered to interact. The application displays the physical bindings between the subunits of a protein complex as a color code matrix where each element represents a binary interaction between two subunits of the complex, and its color shows the best scored interacting domain pair between the subunits, as described in DIMERO (24). Furthermore, for each interaction, all binding domains between the interacting proteins are displayed in a table showing their positions in the sequences and mapping of their residues on the protein structures. Finally, the binding domains are evaluated with DIMERO and the scores measuring the interacting probability are included in the table.

Domain binding sites analysis

The Domain Binding Sites tool is used to display known binding sites of protein domains based on the information contained in 3DID structures. The application offers a user-friendly interface to browse all the interactomics information available in 3DID for the different binding sites of the protein domains. The domain binding sites are clustered according to the number of common interacting amino acids and interface geometry, as described in 3DID (29). The first level of clustering contains interactions that share similar binding sites, whereas in the second level the interactions are grouped in terms of the interacting partner domain family. The binding sites for clusters and domains can be matched and displayed on the current protein structures and sequences; a particular interaction can be retrieved

aligning the domains of the same family at the sequence and structural level (Fig. S3).

Domain-domain template docking

Another tool included in 3DIANA offers the possibility to perform docking between protein structures based on DDI structural templates. To this end, when the application finds structural evidence of an interaction between two domains in different chains, the user can access the different structural conformations of the interacting domain pair. The different structures are clustered according to the geometric conformation of the interfaces as described in 3DID (29). A user-friendly interface allows matching and displaying the binding residues of the DDI templates on the current protein structures and sequences. In addition, a sequence similarity threshold can be set up to filter those DDIs that are under the selected threshold. Indeed, it has been observed that homologous pairs of interacting proteins often interact in the same way, thus, using the same interface. Moreover, above 30% of sequence identity the interface root mean-square deviation decreases significantly and, in general, the higher the sequence identity, the more conserved is the structure of the interaction (48). Thus, we strongly suggest that only templates with at least 30% of sequence similarity should be used to compute DDI template-based docking. However, it must be highlighted that there are also cases in which homologous pairs of domains interact in completely different ways (49). Finally, the application allows docking of two proteins, aligning their domains with the domains of a selected template at structural level (21).

Protein-protein guided docking

3DIANA integrates the PatchDock package (20) to perform protein-guided docking. This tool offers the possibility to model the structure of interacting proteins when knowledge-based information is not available or when the interaction cannot be modeled using domain-domain templates; the individual structures of the interacting proteins are expected to be known. PatchDock uses geometric hashing to maximize the shape complementarity of the subunits, resulting in a highly efficient and fast approach. Finally, the results are scored in terms of the surface complementarity and atomic contact energy (50). PatchDock is a well-known package in the docking field that has been tested in several works, proving its efficiency and performance (51,52). The selection of PatchDock was made for two main reasons. First, it can be guided; thus, the user can select the residues comprising the binding sites. Second, its computational efficiency allows computing solutions in a few minutes or even seconds.

DIANA interface

The web application is designed as a desktop-like environment, where the different tools are organized in individual windows or widgets. The application includes different 3D viewers (53) to display both the structure of the submitted proteins and the DDI structures, easing the browsing of potential binding sites or DDI template structures for protein docking. The application includes a web form that allows the user to define the different subunits of a protein complex combining different structures and chains. Finally, a plugin for Chimera (27) is available to replace the main structural 3D viewer and interface Chimera with the web browser.

Workflow of use

We envision two scenarios for 3DIANA to work for hybrid models. The first one starts from a user-proposed hybrid model and 3DIANA evaluates the probability for the interactions resulting from the proposed hybrid model to be compatible with the current body of known interactomics information. The second application is aimed at helping the researcher to build a hybrid model maximally compatible with known interactomics data, rather than evaluating a proposed one. In the main text of this work we are going to use two different examples. In the first case (911/FEN1 complex), we will present the two types of scenarios indicated previously, whereas the second example (retinoid X receptor (RXR)/vitamin D receptor (VDR) nuclear receptor) will be used to show how 3DIANA models the interaction between two subunits using the template-based docking approach. Several other examples are presented in the Supporting Material.

911/FEN1 complex

Flap endonuclease 1 (FEN1) is a protein involved in DNA replication and repair contributing to maintain the cellular genome integrity. To perform its function, FEN1 is known to associate with the 911 complex, a heterotrimeric ring that encircles DNA and serves as a mobile platform for different enzymes (54–56). The structure of the FEN1/911 complex has not been solved at the atomic level, although the atomic conformation of each of the constituents is known. However, Querol-Audi et al. (57) determined the quaternary structure of the complex at low resolution (18 Å) using single-particle EM of negatively stained samples. Finally, an atomic model was proposed, combining the existing high-resolution structures of the unbound components, which was further refined with molecular dynamics simulations. Fig. 1 presents several views of this complex and the atomic structure of its subunits, with the 911 ring shown in green and FEN1 in red. Note that the three proteins composing the 911 ring are going to be treated as if they were one single subunit, simply to make the interactomics analysis and this presentation easier.

Scenario 1

This case shows how 3DIANA can provide valuable data for the structure modeling of the FEN1/911 complex, exploring all possible DDIs known to exist or predicted by DIMERO between all proteins in the complex. In this way, we have analyzed the structures of the FEN1 protein (PDB: 3Q8K), the 911 heterotrimeric complex (PDB: 3G65), and the FEN1/911 EM volume (Electron Microscopy Data Bank (EMDB): 2029) with 3DIANA through the Chimera plugin. Both subunits are easily

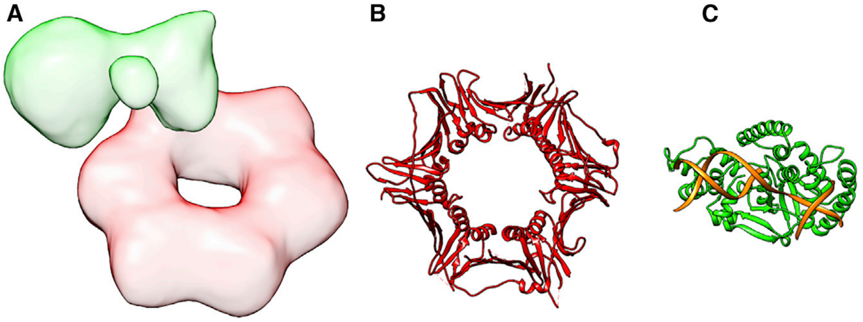

FIGURE 1 Cryo-EM map of the 911/FEN1 complex and atomic structures of its components. (*A*) Cryo-EM density map of the 911/FEN1 complex (EMDB: EMD-2029), in red the volume corresponding to the 911 component and in green the FEN1 subunit. (*B*) Ribbon schema of the 911 complex atomic structure (PDB: 3G65). (*C*) Ribbon schema of the FEN1 atomic structure (PDB: 3Q8K), in orange we show three DNA strands. To see this figure in color, go online.

differentiated in the EM volume (Fig. 1); however, fitting the subunits within the map leads to different possibilities.

The DDI analysis module is useful to explore the known and predicted interaction data between 911 and FEN1 proteins. For this analysis, each of the proteins (911 and FEN1) is decomposed into their Pfam domains, and a multidimensional table of all possible (known and predicted) DDIs is calculated. This large table is presented to the user just focusing on pairs of proteins, indicating if there are structures that are known to contain interacting pairs of domains and, if not, presenting the probability of interaction based on DIMERO scores. Figs. 2 and S2 present one such table and the atomic structure of the best scored domain pairs. Crosses on the right side of the table indicate that the domain pairs cannot be mapped to 3D experimental structures. However, information of predicted DDIs is also presented, and it is indicated with the codes LCP and MCP. The analysis of this table suggests that the most likely DDI happen between domain Rad1 from 911 and xeroderma pigmentosum complementation group G (XPG) from FEN1. In this way, it is immediate to note that the most probable 911 domain interacting with FEN1 should be Rad1, helping to solve the indetermination associated with the low resolution of the map. Thus, based on this information, the Rad1 domain can be placed in the interacting region with FEN1 protein (Fig. 3). Regarding FEN1, we note that there are two XPG domains in FEN1, one at the N-terminal domain and the other toward the center of the protein; both of them were analyzed as candidates to interact with the Rad1 domain of 911.

Once potential interactions between the domains of a given complex have been established, 3DIANA offers two additional modules for those cases in which there are instances of experimental structures relating to these domains: domain binding sites analysis and domain-domain template docking. We have previously indicated that there were no experimental structures involving interactions between the domains of 911 and FEN1, but 3DIANA offers the possibility to focus further the query to concentrate into one of the two interacting domains, exploring which are the known interacting partners for which experimental structures do exist. Focusing on the XPG domains, Fig. S3 presents how the domain binding sites analysis tool of 3DIANA compiles the information on known interacting partners, clustering these interactions into binding sites that are shown along the sequence. Browsing the different binding sites and partners of the XPG domains of FEN1, we found two different types of interacting partners: other members of the XPG family and proliferating cell nuclear antigen (PCNA) domains. Representative examples of binding between domains can be selected. In this way, in Fig. S3 we present the selection of XPG interacting with PCNA, which indeed corresponds to a very similar fold to Rad1, although it does not belong to the same Pfam family. In fact, 3DIANA offers the possibility to superimpose these reported structures onto the hybrid models. In this case, as we indicated before, there was no reported structure between XPG and Rad1 domains; however, 3DIANA is able to partially overlay the structures by aligning the reported interacting pair with respect to XPG. Fig. 4, *A* and *C* show the structural alignment between all possible interacting conformations of XPG and PCNA domains with the FEN1 subunit. Finally, considering the high fold similarity between the PCNA and Rad1 domains, we used the structural matching tool of Chimera to align the conformations displayed in Figs. 4 *A* and 5 *C* with the Rad1 domain of the 911 complex. Fig. 4, *B* and *D*, show the possible conformations of FEN1 and 911 when the XPG and PCNA interacting structures are used as templates to model the interaction.

In this way, 3DIANA has accompanied the user all the way, from having the atomic structure of the individual subunits together with a cryo-EM map

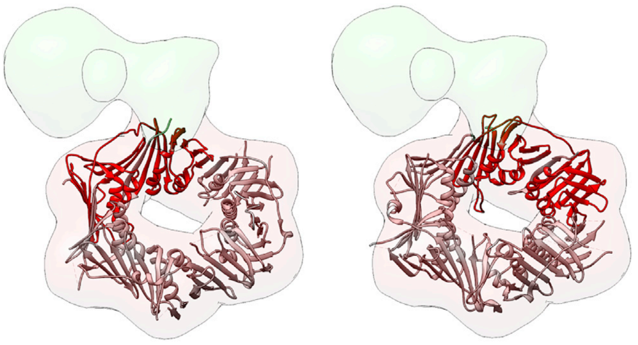

FIGURE 3 Fitting the 911 complex within the cryo-EM density map. Two potential positions of the 911 atomic structure within the EM volume are shown, the Rad1 domain is mapped in red. Note that the two conformations were obtained placing the Rad1 domain (*red ribbons*) next to the volume region corresponding to the FEN1 subunit, assuming that the 911 complex interacts with FEN1 through the Rad1 domain (see *911/FEN1 complex*). To see this figure in color, go online.

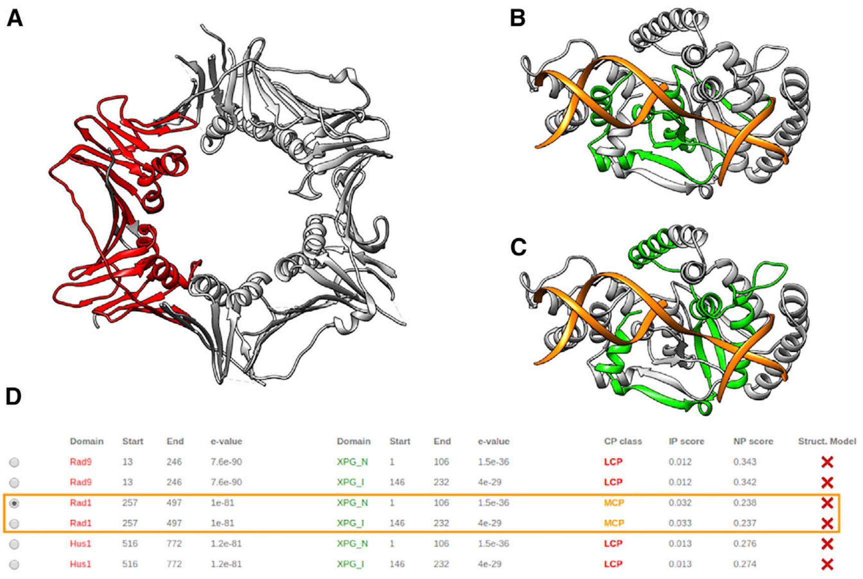

	Domain	Start	End	e-value	Domain	Start	End	e-value	CP class	IP score	NP score	Struct. Model
○	Rad9	13	246	7.6e-90	XPG_N	1	106	1.5e-36	LCP	0.012	0.343	✗
○	Rad9	13	246	7.6e-90	XPG_I	146	232	4e-29	LCP	0.012	0.342	✗
◉	Rad1	257	497	1e-81	XPG_N	1	106	1.5e-36	MCP	0.032	0.238	✗
○	Rad1	257	497	1e-81	XPG_I	146	232	4e-29	MCP	0.033	0.237	✗
○	Hus1	516	772	1.2e-81	XPG_N	1	106	1.5e-36	LCP	0.013	0.276	✗
○	Hus1	516	772	1.2e-81	XPG_I	146	232	4e-29	LCP	0.013	0.274	✗

FIGURE 2 3DIANA DDI analysis. (*A*) In red, mapping of the Rad1 domain in the 911 complex structure. (*B*) Green, mapping of the XPG_I domain in the FEN1 protein structure; orange, three strands of DNA. (*C*) Green, mapping of the XPG_N domain in the FEN1 protein structure; orange, three strands of DNA. (*D*) The DDI table shows the probability of interaction between the domain pairs of the selected subunits (911 and FEN1); note that the best scored domain pairs (Rad1-XPG_N and Rad1-XPG_I) are shown within the orange rectangle. To see this figure in color, go online.

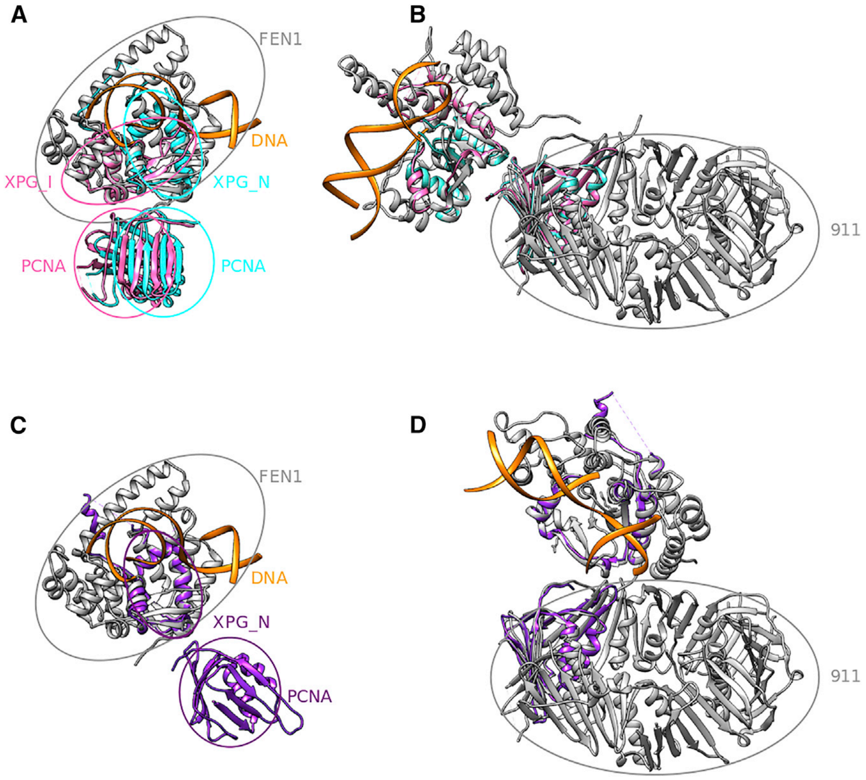

FIGURE 4 Structural alignment of DDI templates. (*A*) Structural alignment between FEN1 (*gray*) and the DDI templates XPG_N-PCNA (*cyan*) and XPG_I-PCNA (*pink*), extracted from the PDB: 1UL1 structure. (*B*) Structural alignment of the PCNA domain contained in the templates shown in (*A*) (*cyan* and *pink structures*) with the Rad1 domain of the 911 complex. The FEN1 structure is aligned to the XPG domain of the templates. Only one conformation is displayed because both templates lead to very similar results. (*C*) Structural alignment between FEN1 (*gray*) protein and the DDI templates XPG_N-PCNA (*purple*) collected from the PDB: 1UL1 structure; note that this template leads to an architecture of the complex that is different from the one shown in (*B*), which is not supported by the cryo-EM data. (*D*) Structural alignment of the PCNA domain contained in the template exposed in (*C*) (*purple structure*) with the Rad1 domain of the 911 complex. The FEN1 structure is aligned to the XPG domain of the template. To see this figure in color, go online.

of the complex, to providing templates of DDIs and, finally, building potential models of the complex. Clearly, 3DIANA has not been designed to build structural conformations close to satisfying the physical restrains of interactions between proteins. Indeed, it aims at proposing initial models that could be further refined by other approaches, such as docking algorithms (22,58), molecular dynamics methods (12,26,59,60), or flexible fitting (10,61).

Scenario 2

In this case a given hybrid model is proposed by the user without using 3DIANA and then, 3DIANA evaluates its likelihood in terms of the DDIs implied in the proposed hybrid model. In the case of EMDB: 2029, the original authors did not provide a fitting model (although in the body of the work a given set of interactions is proposed), but for the sake of argument we would consider that two possible hybrid models could have been submitted, shown in Fig. 5, *A* and *B*. At this stage 3DIANA evaluates the physical bindings in terms of the DDIs implied by the models; this information is displayed in a matrix where the different elements represent physical bindings between different subunits (see Figs. 5, *A* and *B*, and S1, *A* and *B*). Analyzing the two matrices of interactions for the two pseudohybrid models, it is clear that the one in Fig. 5 *A* has interactions that are at least MCPs, whereas the model in Fig. 5 *B* at most has LCPs. On the basis of this information, the first model would

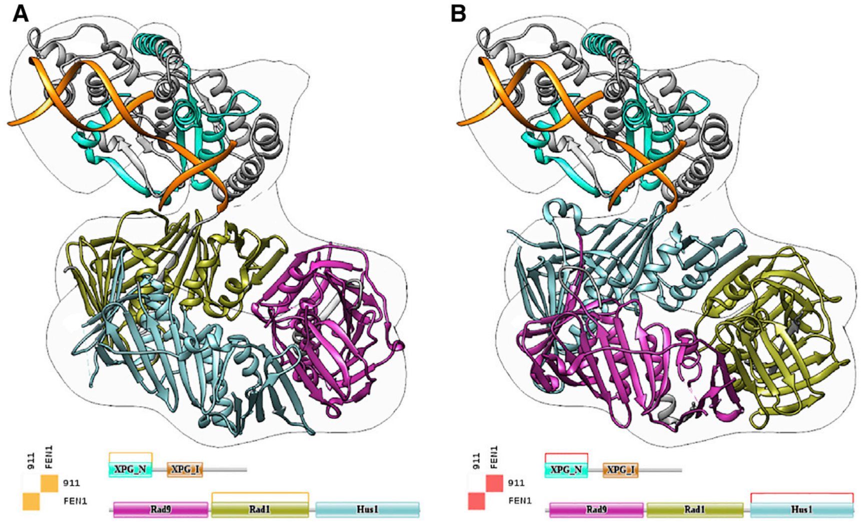

FIGURE 5 3DIANA model evaluation with DIMERO scores. (*A*) Possible structure of the 911/FEN1 complex fitted in its cryo-EM map. In this configuration, the binding between the subunits occurs through the XPG_N domain of FEN1 and the Rad1 domain of 911. The valuation using DIMERO scores of the physical interactions between the subunits and domains in the proposed model is displayed behind the structure, showing how the interaction between the XPG_N domain of FEN1 and Rad1 of 911 is scored as medium confidence prediction (MCP; *orange*). (*B*) A different alternative model of the 911/FEN1 complex fitted in the same cryo-EM map. The binding between the subunits occurs through the XPG_N domain of FEN1 and the Hus1 domain of 911. In this case, the interaction is scored as low confidence prediction (LCP; *red*). To see this figure in color, go online.

be preferred over the second and this was, indeed, the one proposed by the authors.

RXR/VDR nuclear receptor

This example shows how 3DIANA can be used to model interactions between subunits in a given complex performing template-based docking through solved DDI structures. Orlov et al. (62) determined the structure of the RXR/VDR nuclear receptor at low resolution (12 Å) using single-particle cryo-EM (EMDB: 1985). The RXR and VDR subunits are composed of two well-conserved core domains, the DNA-binding domain and the ligand-binding domain (LBD). To display the different domains of the RXR and VDR subunits and provide a more detailed representation of the complex, the authors fitted the available crystal structures of the LBDs and DNA-binding domains. In particular, the interaction between the LBD domains of the RXR/VDR dimer was modeled with the structure of the RXR/RAR complex (PDB: 1DKF (63)), replacing the retinoic acid receptor structure by the VDR-LBD subunit (PDB: 1DB1 (64)) through structural alignment. 3DIANA was designed to facilitate this type of modeling through template-based docking, and to show the power of the platform, in this example we will focus on the modeling of the LBD domains of the RXR and VDR proteins.

For this demonstration, we have used the same VDR-LBD structure from the PDB: 1DB1 but, instead of using the RXR-LBD mouse protein of the PDB: 1DKF, we selected the human RXR subunit of the PDB: 2P1T structure (65) because the complex in the original study consisted of human proteins. Both structures were opened in Chimera and 3DIANA was started. In the first step, we used the DDI analysis tool to explore the predicted and knowledge-based DDI information available to model the interaction between RXR and VDR. Fig. 6 displays the LBD domains (Pfam domain name: Hormone_recep) of the RXR and VDR structures and the results of the analysis; the DIMERO score is MCP and the green tick under the Struct. Model column indicates that DDI templates are available for template-based modeling. We then decided to use the Domain-Domain Template Docking to explore the possible domain pairs for which structural templates are available. Fig. S4 shows the graphical interface for the domain pair selection, in this example both proteins contain a single domain; however; if multiple combinations were available, the user could select the preferred domain interaction to be modeled. The sequence identity field (Fig. S4, *red box*) allows the user to select only those DDI templates above a certain threshold of sequence identity; thus, the sequence identity between the template domains and the particular domains to be modeled are above the selected threshold. By default, this option is set up at 30%, because it has been shown that above this threshold the interface root mean-square deviation decreases significantly (48). In this example, we used the default option and the possible DDI templates were fetched. Fig. S5 displays the graphical interface to explore the different templates available for the selected pair of domains and sequence identity threshold. The DDI templates are organized in clusters grouped by the interface conformation (Fig. S5 *A*; see *DDI analysis*). The cluster panel also shows the highest sequence identity value of the cluster templates calculated against the domains to be modeled. We selected the cluster and DDI template with the highest sequence identity (PDB: 1XV9). When selected, the structure of the template is displayed in a 3D viewer (Fig. S5 *D*) and its sequences are aligned with the domains of interest highlighting the interacting amino acids (Fig. S5, *B* and *C*). Furthermore, the interacting amino acids are also mapped in the complex subunits and displayed in the main 3D viewer (Fig. 7 *A*); in this manner the interacting residues defined by the particular DDI template can be checked before docking. Once the desired DDI template is selected, 3DIANA can perform docking by aligning the subunits to the template. Fig. 7 *B* shows the model structure of the RXR-LBD/VDR-LBD based on the LBD-LBD template contained in PDB: 1XV9. Finally, this model was manually fitted in the cryo-EM map (EMDB: 1985; Fig. 7 *C*) to check if the proposed conformation was compatible with the map, with good results.

RESULTS AND DISCUSSION

In this work we present 3DIANA, a novel web platform that interfaces EM hybrid models with DDI predictions as well as with current knowledge of experimentally determined protein structures, aimed at guiding the quaternary structure modeling of protein complexes from medium and low resolution structural data. A current amount of interactomics data have been proven to be efficient predicting interactions between protein domains (24) and modeling the structure of PPIs based on DDI structural templates (25). Most of the integrated approaches in 3DIANA are based on statistical methods providing fast predictions of preliminary models. Initial predictions can be refined using more sophisticated algorithms based on structure geometry or physicochemical approaches and leading to more accurate results. Furthermore, the architecture of 3DIANA allows the integration of new packages and in future versions new ab initio methods and refinement tools will be available in the platform.

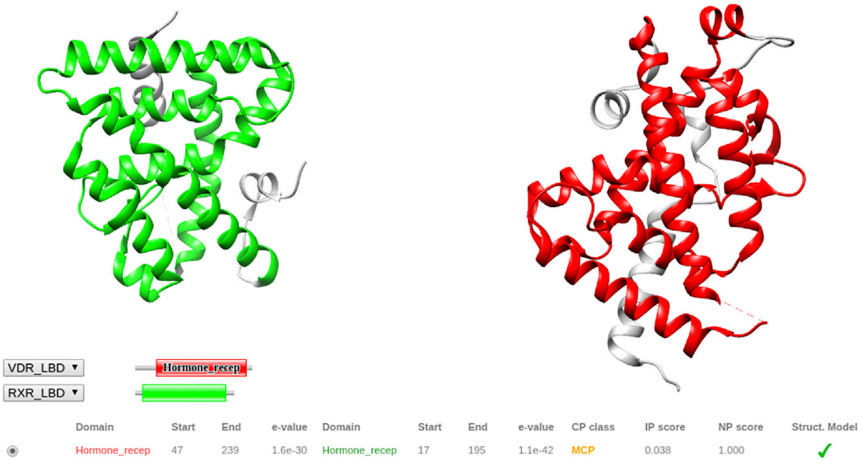

FIGURE 6 RXR and VDR DDI analysis. Green, LBD domain (Pfam name: Hormone_recep) of RXR protein (PDB: 2PT1); red, LBD domain (Pfam name: Hormone_recep) of VDR protein (PDB: 1DB1). (*Bottom*) 3DIANA analysis of the domain pair Hormone_recep-Hormone_recep, showing that the DIMERO interacting score corresponds to MCP (medium confidence prediction); the green tick on the Struct. Model column indicates that structural templates are available to model the interaction between the domains. To see this figure in color, go online.

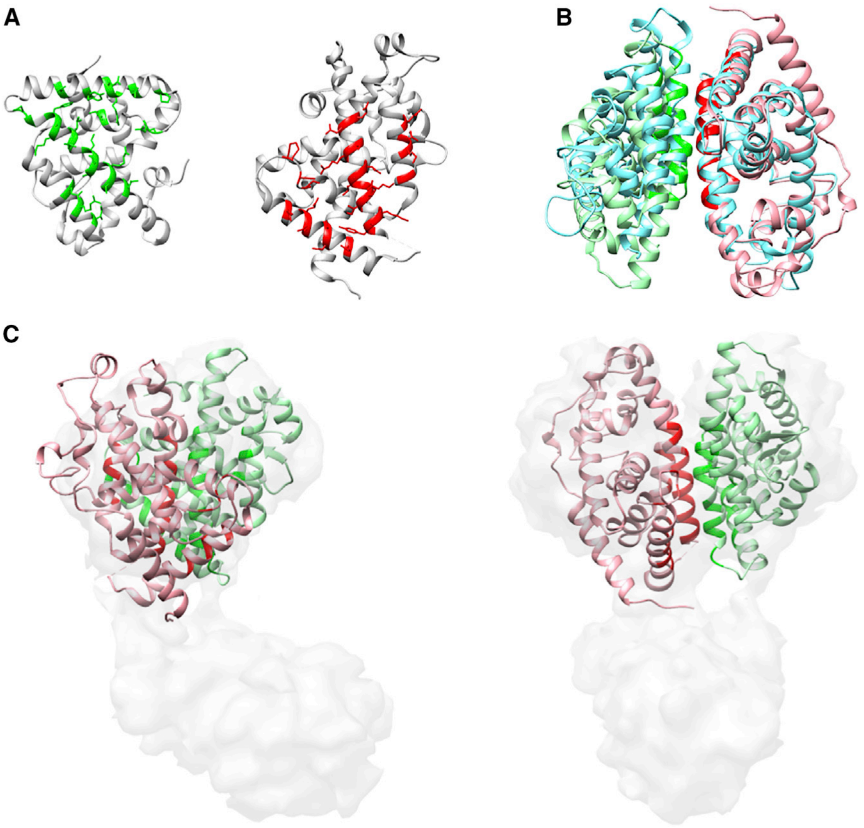

FIGURE 7 Modeling of the RXR-LBD/VDR-LBD interaction. (*A*) Structures of the RXR-LDB domain (PDB: 2PT1; *left*) and VDR-LDB domain (PDB: 1DB1; *right*). In green and red, we represent the mapping of the interacting residues when the PDB: 1XV9 structure is used to model the interaction. (*B*) Template-based docking of the RXR-LDB and VDR-LDB domains when the PDB: 1XV9 structure (*cyan*) is used as a template. (*C*) Manual fitting of the RXR-LBD/VDR-LBD model in the RXR/VDR EM map (EMDB: 1985). To see this figure in color, go online.

Currently, the application can perform different types of analysis and calculations. It allows assessment of potential physical contacts between protein domains, providing an independent measure to evaluate a given model when the user has been able to place the different components within the density map using other sources of information or approaches. In addition, 3DIANA can be used to enrich structural data, annotating experimentally determined biding sites, or modeling the structure of interactions using domain-domain templates. Alternatively, when no information exists on how components could fit or interact because no experimental data is available or the resolution of the map is not enough to place the different subunits, the strategy of DDI prediction can be used to determine which regions of the different interacting proteins may be involved in physical bindings.

3DIANA is accessible through a web browser at http://3diana.cnb.csic.es or through Chimera 3D viewer installing the 3DIANA Chimera plugin. All tools integrated in the platform offer a user-friendly and intuitive interface to compute the different scores or to browse the different sources of information; an online help is always available for all widgets. When the Chimera plugin is not used, the web interface integrates Jsmol to display the different 3D structures.

Finally, we would like to point out that 3DIANA has been designed to provide a first approximation of how subunits could interact and fit within a density map. For that reason the methodologies integrated in the platform are highly efficient in terms of computational cost but not as accurate as other approaches that have been designed to maximize the modeling accuracy (22,66,67). For that reason, we suggest that models obtained with 3DIANA should be refined using highly accurate tools. For example, EMfit could be used to refine manual fittings performed by the users, the Rosetta package (8) offers different protocols to refine the structure of interacting proteins, or FireDock (68) is a method to apply backbone flexibility to the interface of proteins.

SUPPORTING MATERIAL

Supporting Materials and Methods and eight figures are available at http://www.biophysj.org/biophysj/supplemental/S0006-3495(15)04703-7.

AUTHOR CONTRIBUTIONS

J.S.: concept, design, acquisition, analysis and interpretation of data, and writing of the manuscript. R.S.G.: design and analysis of data. D.T.M.: design and analysis of data. J.C.A.: design and analysis of data. C.O.S.S.: concept, supervision, analysis, and interpretation of data, and writing of the article. J.M.C.: concept, supervision, analysis and interpretation of data, and writing of the article. All authors read and approved the final manuscript.

ACKNOWLEDGMENTS

This work was supported by the Instituto de Salud Carlos III, project number PT13/0001/0009 funding the Spanish National Institute of Bioinformatics, the Spanish Ministry of Economy and Competitiveness through grants AIC-A-2011-0638 and BIO2013-44647-R and the European Union (EU) and Horizon 2020 through grant CORBEL (INFRADEV-1-2014-1 - Proposal: 654248). C.O.S.S. is recipient of a Ramón y Cajal fellowship. J.S. is recipient of a Juan de la Cierva fellowship.

REFERENCES

1. Nogales, E., and S. H. Scheres. 2015. Cryo-EM: a unique tool for the visualization of macromolecular complexity. *Mol. Cell.* 58:677–689.
2. Liao, M., E. Cao, …, Y. Cheng. 2013. Structure of the TRPV1 ion channel determined by electron cryo-microscopy. *Nature.* 504:107–112.
3. Bai, X. C., I. S. Fernandez, …, S. H. Scheres. 2013. Ribosome structures to near-atomic resolution from thirty thousand cryo-EM particles. *eLife.* 2:e00461.
4. Lu, P., X. C. Bai, …, Y. Shi. 2014. Three-dimensional structure of human γ-secretase. *Nature.* 512:166–170.
5. Lindert, S., N. Alexander, …, J. Meiler. 2012. EM-fold: de novo atomic-detail protein structure determination from medium-resolution density maps. *Structure.* 20:464–478.
6. Baker, M. L., M. R. Baker, …, W. Chiu. 2012. Gorgon and pathwalking: macromolecular modeling tools for subnanometer resolution density maps. *Biopolymers.* 97:655–668.
7. Topf, M., M. L. Baker, …, A. Sali. 2006. Refinement of protein structures by iterative comparative modeling and Cryo-EM density fitting. *J. Mol. Biol.* 357:1655–1668.
8. DiMaio, F., M. D. Tyka, …, D. Baker. 2009. Refinement of protein structures into low-resolution density maps using rosetta. *J. Mol. Biol.* 392:181–190.
9. Tjioe, E., K. Lasker, …, A. Sali. 2011. MultiFit: a web server for fitting multiple protein structures into their electron microscopy density map. *Nucleic Acids Res.* 39:W167–W170.
10. Woetzel, N., S. Lindert, …, J. Meiler. 2011. BCL:EM-Fit: rigid body fitting of atomic structures into density maps using geometric hashing and real space refinement. *J. Struct. Biol.* 175:264–276.
11. Saha, M., and M. C. Morais. 2012. FOLD-EM: automated fold recognition in medium- and low-resolution (4-15 Å) electron density maps. *Bioinformatics.* 28:3265–3273.
12. DiMaio, F., Y. Song, …, D. Baker. 2015. Atomic-accuracy models from 4.5-Å cryo-electron microscopy data with density-guided iterative local refinement. *Nat. Methods.* 12:361–365.
13. Wang, R. Y., M. Kudryashev, …, F. DiMaio. 2015. De novo protein structure determination from near-atomic-resolution cryo-EM maps. *Nat. Methods.* 12:335–338.
14. Sorzano, C. O. S., J. Vargas, …, J. M. Carazo. 2015. Fast and accurate conversion of atomic models into electron density maps. *AIMS Biophysics.* 2:8–20.
15. Vasishtan, D., and M. Topf. 2011. Scoring functions for cryo-EM density fitting. *J. Struct. Biol.* 174:333–343.
16. de Vries, S. J., and M. Zacharias. 2012. ATTRACT-EM: a new method for the computational assembly of large molecular machines using cryo-EM maps. *PLoS One.* 7:e49733.
17. Zacharias, M. 2005. ATTRACT: protein-protein docking in CAPRI using a reduced protein model. *Proteins.* 60:252–256.
18. Lasker, K., A. Sali, and H. J. Wolfson. 2010. Determining macromolecular assembly structures by molecular docking and fitting into an electron density map. *Proteins.* 78:3205–3211.
19. Duhovny, D., R. Nussinov, and H. J. Wolfson. 2002. Efficient unbound docking of rigid molecules. *In* Algorithms in Bioinformatics. R. Guigó and D. Gusfield, editors. Springer, pp. 185–200.
20. Rossmann, M. G. 2000. Fitting atomic models into electron-microscopy maps. *Acta Crystallogr. D Biol. Crystallogr.* 56:1341–1349.
21. van Zundert, G. C., A. S. Melquiond, and A. M. Bonvin. 2015. Integrative modeling of biomolecularcComplexes: HADDOCKing with cryo-electron microscopy data. *Structure.* 23:949–960.
22. Dominguez, C., R. Boelens, and A. M. Bonvin. 2003. HADDOCK: a protein-protein docking approach based on biochemical or biophysical information. *J. Am. Chem. Soc.* 125:1731–1737.
23. Ludtke, S. J., C. L. Lawson, …, W. Chiu. 2012. The 2010 cryo-EM modeling challenge. *Biopolymers.* 97:651–654.
24. Segura, J., C. O. Sorzano, …, J. M. Carazo. 2015. Using neighborhood cohesiveness to infer interactions between protein domains. *Bioinformatics.* 31:2545–2552.
25. Mosca, R., A. Céol, and P. Aloy. 2013. Interactome3D: adding structural details to protein networks. *Nat. Methods.* 10:47–53.
26. Vagin, A. A., R. A. Steiner, …, G. N. Murshudov. 2004. REFMAC5 dictionary: organization of prior chemical knowledge and guidelines for its use. *Acta Crystallogr. D Biol. Crystallogr.* 60:2184–2195.
27. Pettersen, E. F., T. D. Goddard, …, T. E. Ferrin. 2004. UCSF Chimera–a visualization system for exploratory research and analysis. *J. Comput. Chem.* 25:1605–1612.
28. Ghoorah, A. W., M. D. Devignes, …, D. W. Ritchie. 2013. Protein docking using case-based reasoning. *Proteins.* 81:2150–2158.
29. Mosca, R., A. Céol, …, P. Aloy. 2014. 3did: a catalog of domain-based interactions of known three-dimensional structure. *Nucleic Acids Res.* 42:D374–D379.
30. Sillitoe, I., T. E. Lewis, …, C. A. Orengo. 2015. CATH: comprehensive structural and functional annotations for genome sequences. *Nucleic Acids Res.* 43:D376–D381.
31. Andreeva, A., D. Howorth, …, A. G. Murzin. 2014. SCOP2 prototype: a new approach to protein structure mining. *Nucleic Acids Res.* 42:D310–D314.
32. Mitchell, A., H. Y. Chang, …, R. D. Finn. 2015. The InterPro protein families database: the classification resource after 15 years. *Nucleic Acids Res.* 43:D213–D221.
33. Sigrist, C. J., E. de Castro, …, I. Xenarios. 2013. New and continuing developments at PROSITE. *Nucleic Acids Res.* 41:D344–D347.
34. Finn, R. D., A. Bateman, …, M. Punta. 2014. Pfam: the protein families database. *Nucleic Acids Res.* 42:D222–D230.
35. Johnson, L. S., S. R. Eddy, and E. Portugaly. 2010. Hidden Markov model speed heuristic and iterative HMM search procedure. *BMC Bioinformatics.* 11:431.
36. Itzhaki, Z., E. Akiva, …, H. Margalit. 2006. Evolutionary conservation of domain-domain interactions. *Genome Biol.* 7:R125.
37. Riley, R., C. Lee, …, D. Eisenberg. 2005. Inferring protein domain interactions from databases of interacting proteins. *Genome Biol.* 6:R89.
38. Nye, T. M., C. Berzuini, …, S. A. Teichmann. 2005. Statistical analysis of domains in interacting protein pairs. *Bioinformatics.* 21:993–1001.
39. Liu, Y., N. Liu, and H. Zhao. 2005. Inferring protein-protein interactions through high-throughput interaction data from diverse organisms. *Bioinformatics.* 21:3279–3285.
40. Ng, S. K., Z. Zhang, and S. H. Tan. 2003. Integrative approach for computationally inferring protein domain interactions. *Bioinformatics.* 19:923–929.
41. Gomez, S. M., W. S. Noble, and A. Rzhetsky. 2003. Learning to predict protein-protein interactions from protein sequences. *Bioinformatics.* 19:1875–1881.
42. Deng, M., S. Mehta, …, T. Chen. 2002. Inferring domain-domain interactions from protein-protein interactions. *Genome Res.* 12:1540–1548.
43. Sprinzak, E., and H. Margalit. 2001. Correlated sequence-signatures as markers of protein-protein interaction. *J. Mol. Biol.* 311:681–692.
44. Szklarczyk, D., A. Franceschini, …, C. von Mering. 2015. STRING v10: protein-protein interaction networks, integrated over the tree of life. *Nucleic Acids Res.* 43:D447–D452.

45. Rose, P. W., C. Bi, …, P. E. Bourne. 2013. The RCSB Protein Data Bank: new resources for research and education. *Nucleic Acids Res.* 41:D475–D482.

46. Aloy, P., and R. B. Russell. 2004. Ten thousand interactions for the molecular biologist. *Nat. Biotechnol.* 22:1317–1321.

47. Kim, W. K., A. Henschel, …, M. Schroeder. 2006. The many faces of protein-protein interactions: a compendium of interface geometry. *PLOS Comput. Biol.* 2:e124.

48. Aloy, P., H. Ceulemans, …, R. B. Russell. 2003. The relationship between sequence and interaction divergence in proteins. *J. Mol. Biol.* 332:989–998.

49. Park, S. Y., B. D. Beel, …, B. R. Crane. 2004. In different organisms, the mode of interaction between two signaling proteins is not necessarily conserved. *Proc. Natl. Acad. Sci. USA.* 101:11646–11651.

50. Zhang, C., G. Vasmatzis, …, C. DeLisi. 1997. Determination of atomic desolvation energies from the structures of crystallized proteins. *J. Mol. Biol.* 267:707–726.

51. Segura, J., M. A. Marín-López, …, N. Fernandez-Fuentes. 2015. VORFFIP-driven dock: V-D2OCK, a fast and accurate protein docking strategy. *PLoS One.* 10:e0118107.

52. Amir, N., D. Cohen, and H. J. Wolfson. 2015. DockStar: a novel ILP-based integrative method for structural modeling of multimolecular protein complexes. *Bioinformatics.* 31:2801–2807.

53. Jmol. 2013. Jmol: an open-source Java viewer for chemical structures in 3D. http://jmol.sourceforge.net/. Accessed December 29, 2015.

54. Doré, A. S., M. L. Kilkenny, …, L. H. Pearl. 2009. Crystal structure of the rad9-rad1-hus1 DNA damage checkpoint complex–implications for clamp loading and regulation. *Mol. Cell.* 34:735–745.

55. Sohn, S. Y., and Y. Cho. 2009. Crystal structure of the human rad9-hus1-rad1 clamp. *J. Mol. Biol.* 390:490–502.

56. Xu, M., L. Bai, …, T. Jiang. 2009. Structure and functional implications of the human rad9-hus1-rad1 cell cycle checkpoint complex. *J. Biol. Chem.* 284:20457–20461.

57. Querol-Audí, J., C. Yan, …, I. Ivanov. 2012. Repair complexes of FEN1 endonuclease, DNA, and Rad9-Hus1-Rad1 are distinguished from their PCNA counterparts by functionally important stability. *Proc. Natl. Acad. Sci. USA.* 109:8528–8533.

58. Gray, J. J., S. Moughon, …, D. Baker. 2003. Protein-protein docking with simultaneous optimization of rigid-body displacement and side-chain conformations. *J. Mol. Biol.* 331:281–299.

59. Trabuco, L. G., E. Villa, …, K. Schulten. 2008. Flexible fitting of atomic structures into electron microscopy maps using molecular dynamics. *Structure.* 16:673–683.

60. Trabuco, L. G., E. Villa, …, K. Schulten. 2009. Molecular dynamics flexible fitting: a practical guide to combine cryo-electron microscopy and X-ray crystallography. *Methods.* 49:174–180.

61. Topf, M., K. Lasker, …, A. Sali. 2008. Protein structure fitting and refinement guided by cryo-EM density. *Structure.* 16:295–307.

62. Orlov, I., N. Rochel, …, B. P. Klaholz. 2012. Structure of the full human RXR/VDR nuclear receptor heterodimer complex with its DR3 target DNA. *EMBO J.* 31:291–300.

63. Bourguet, W., V. Vivat, …, D. Moras. 2000. Crystal structure of a heterodimeric complex of RAR and RXR ligand-binding domains. *Mol. Cell.* 5:289–298.

64. Rochel, N., J. M. Wurtz, …, D. Moras. 2000. The crystal structure of the nuclear receptor for vitamin D bound to its natural ligand. *Mol. Cell.* 5:173–179.

65. Koropatkin, N., A. M. Randich, …, T. J. Smith. 2007. The structure of the iron-binding protein, FutA1, from Synechocystis 6803. *J. Biol. Chem.* 282:27468–27477.

66. DiMaio, F., A. Leaver-Fay, …, I. André. 2011. Modeling symmetric macromolecular structures in Rosetta3. *PLoS One.* 6:e20450.

67. Sircar, A., S. Chaudhury, …, J. J. Gray. 2010. A generalized approach to sampling backbone conformations with RosettaDock for CAPRI rounds 13-19. *Proteins.* 78:3115–3123.

68. Andrusier, N., R. Nussinov, and H. J. Wolfson. 2007. FireDock: fast interaction refinement in molecular docking. *Proteins.* 69:139–159.

Biophysical Journal, Volume *110*

Supplemental Information

3DIANA: 3D Domain Interaction Analysis: A Toolbox for Quaternary Structure Modeling

Joan Segura, Ruben Sanchez-Garcia, Daniel Tabas-Madrid, Jesus Cuenca-Alba, Carlos Oscar S. Sorzano, and Jose Maria Carazo

Biophysical Journal

Supporting Material

3DIANA: 3D Domain Interaction Analysis: A Toolbox for Quaternary Structure Modeling

Joan Segura,[1,*] Ruben Sanchez-Garcia,[1] Daniel Tabas-Madrid,[1] Jesus. Cuenca-Alba,[1] Carlos Oscar S. Sorzano,[1] and Jose Maria Carazo[1]

[1]GN7, Spanish National Institute for Bioinformatics (INB) and Biocomputing Unit, National Center of Biotechnology (CSIC)/Instruct Image Processing Center, Madrid, Spain

S1 3DIANA web interfaces

This section presents the different graphical interfaces of the different tools integrated in 3DIANA web platform (see Section 2.2 in the main manuscript).

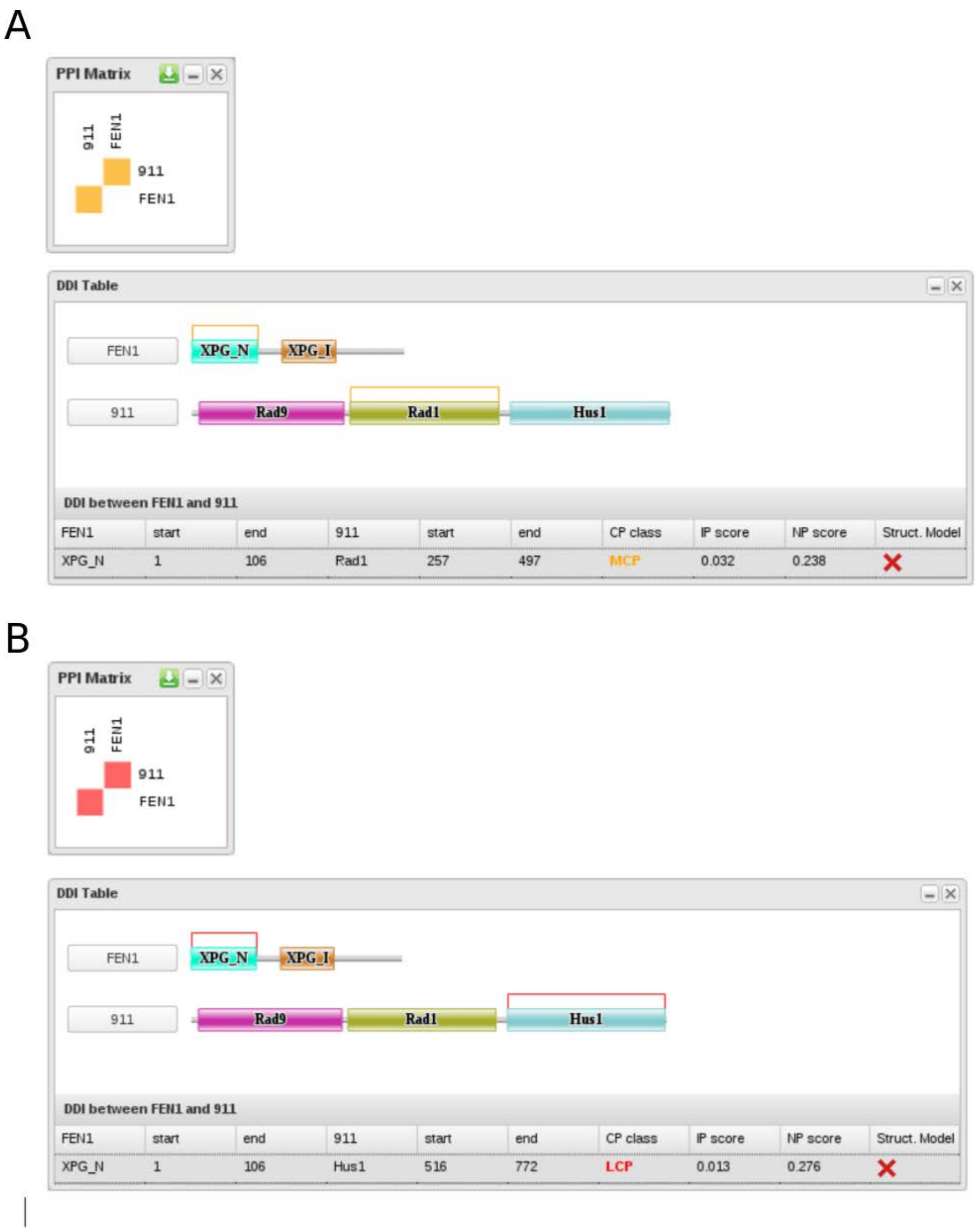

Figure S1: 3DIANA 'Domain-Domain Binding Analysis' tool. (A) Evaluation of the physical interactions between the subunits and domains in the proposed model (see Figure 2A main manuscript). The PPI matrix in the upper left corner displays the possible physical interactions between the different subunits, in this case 911 and FEN1, and the color indicates the best scored interaction between the domains of the two subunits (orange corresponds to medium confidence predictions –MCP-). Interactions between domains are displayed in a table with their corresponding DIMERO scores. In this case, the interaction is between the XPG_N domain of FEN1 and Rad1 of 911 and the interaction is scored as MCP. (B) Evaluation of a different proposed model for the 911/FEN1 complex (see Figure 2B in main text). In this case, the interaction is between the XPG_N domain of FEN1 and hus1 of 911, and the interaction is scored as low confidence prediction (LCP); note that this possible interaction between the two subunits is displayed in red in the PPI matrix.

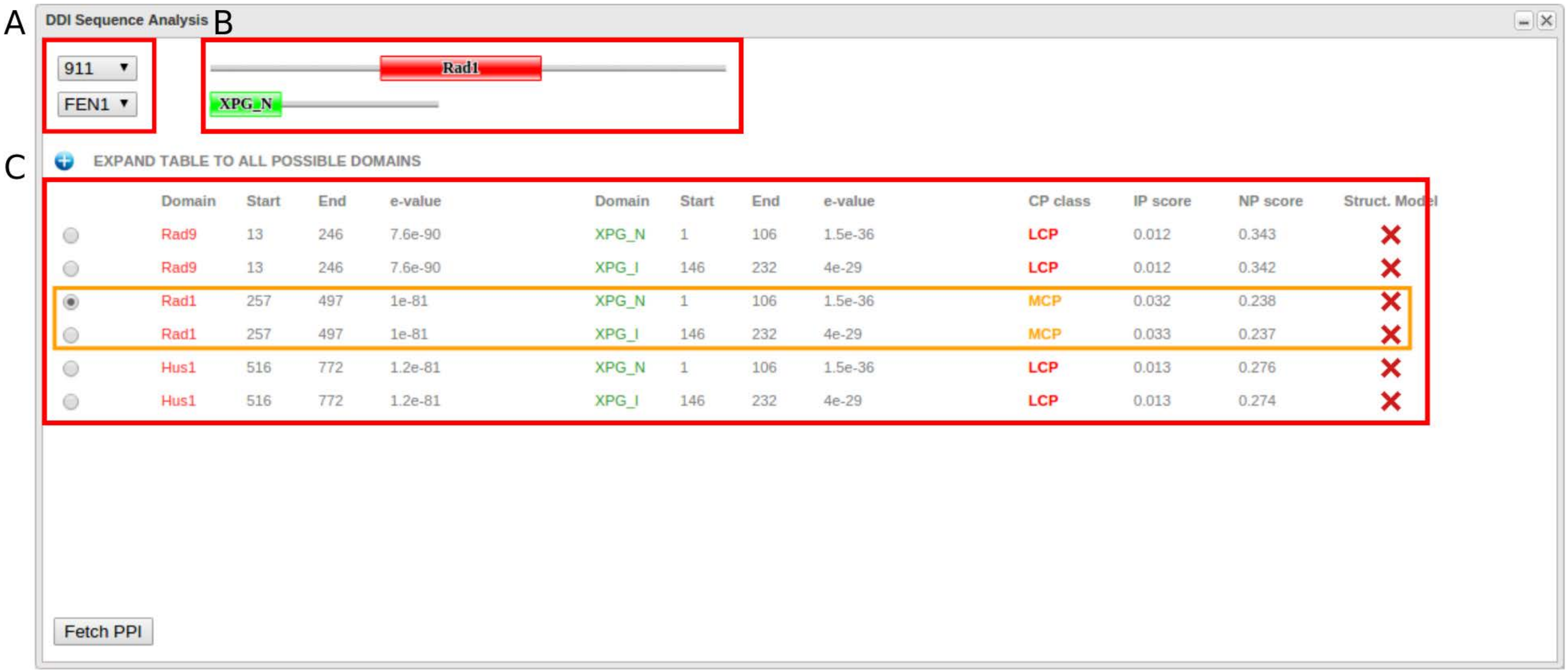

Figure S2: 3DIANA 'Domain-Domain Interacting Analysis' tool. 3DIANA GUI of the 'Domain-Domain Interacting Analysis' tool. (A) Selected pair of subunits in the analysis, in this example the 911 and the FEN1 subunits. (B) Graphical schema of the selected domain pairs (Rad1-XPG_N) in their respective sequences. (C) Domain-domain interaction table evaluating the probability of interaction between the domain pairs of the selected subunits (911 and FEN1); note that the best scored domain pairs (Rad1-XPG_N and Rad1-XPG_I) are shown within the orange rectangle)

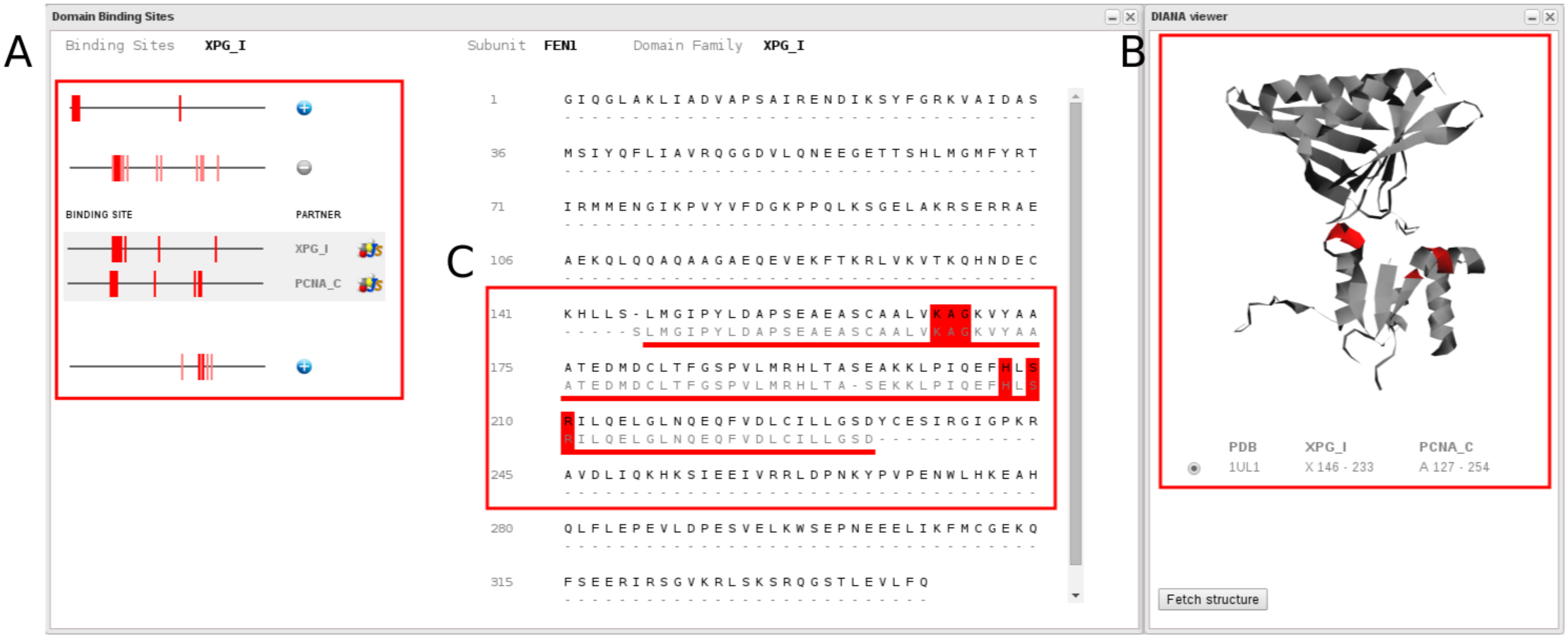

Figure S3: 3DIANA GUI of the 'Domain Binding Sites Analysis' tool. (A) Clustering of the different binding sites of the XPG_I domain family, the domain sequence is represented with a black line and the interacting residues of the binding sites are marked with perpendicular red segments. Each of the clusters is defined by different interactions between XPG_I domains and other domain partners. (B) Mapping of the potential interacting residues of XPG_I domain in FEN1 when XPG_I interacts with a PCNA domain, this information is derived from the DDI template contained in the PDB structure 1UL1; note that the alignment between both XPG_I (FEN1 and DDI template) domains is also displayed. (C) Structural viewer displaying the selected interactions, in this example the interaction between the XPG_I and PCNA domains contained in the PDB structure 1UL1.

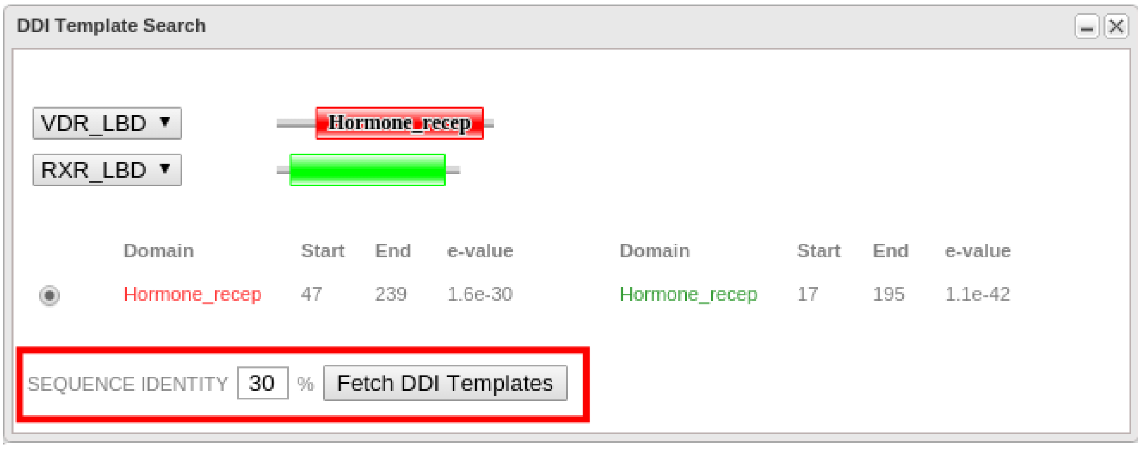

Figure S4: Domain-Domain Template Selection Interface. The interface allows to explore the available DDI templates for modeling the structure of the domains contained in the selected subunits. A threshold can be set up to filter those templates under a certain degree of sequence identity with the domains of interest.

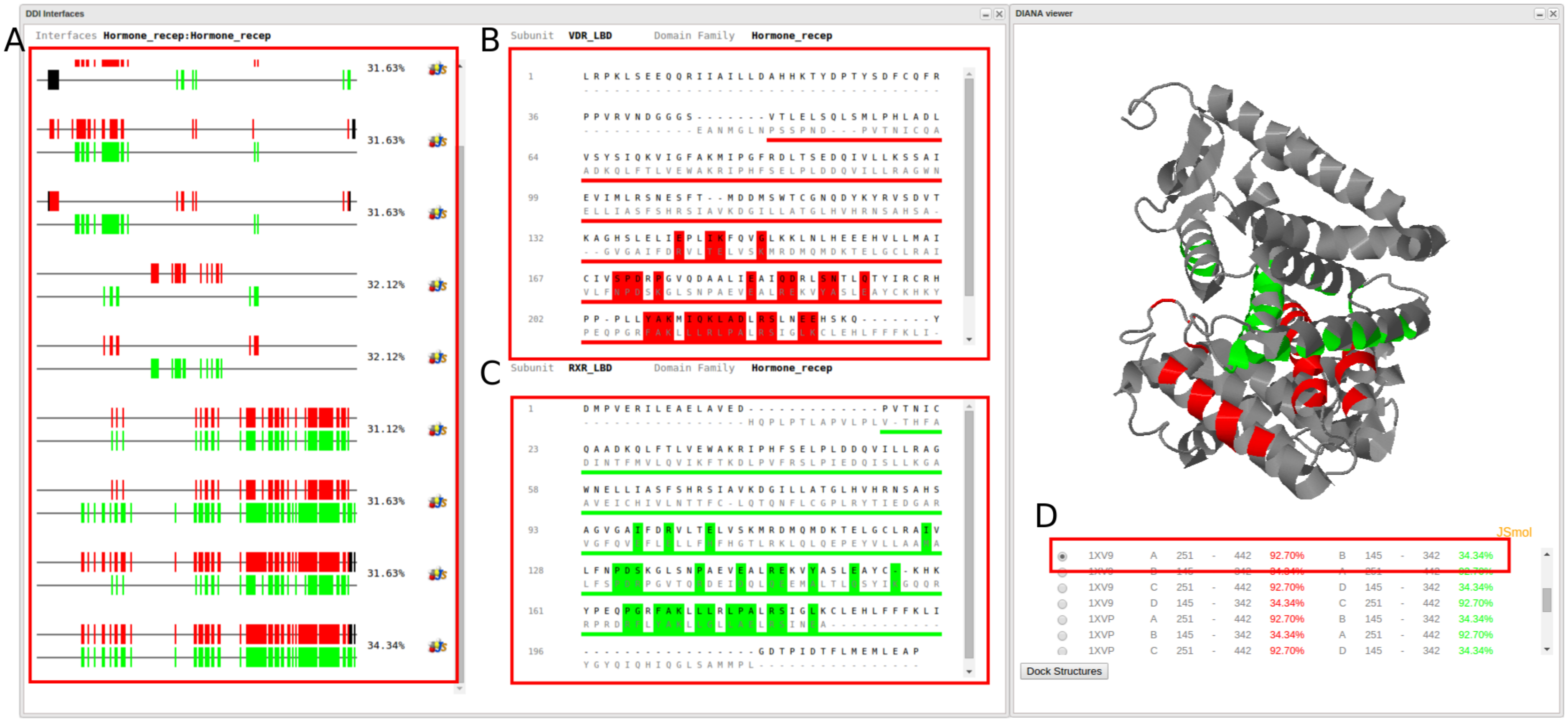

Figure S5: Domain-Domain Interaction Interfaces. (A) Templates are organized in clusters according to their interface conformation (see Section 2.1), also the highest sequence identity value of the templates compared with the domains of interest is displayed on the right. The interacting residues are represented in green and red segments along the domains sequence. (B,C) Sequence alignment of the selected template domains with the domains of interest underlined with a red and green lines. The interacting amino acids are highlighted in red and green colors. (D) 3D conformation of the selected DDI template, the interacting residues are mapped in red and green colors**.**

SM2 3DIANA examples using the 'Domain-Domain Binding Analysis' tool

In this section we present different cases of atomic structures solved by means of cryoEM and the corresponding results obtained with the 'Domain-Domain Binding Analysis' tool. The purpose of these analyses is to show how DIMERO predictions are in agreement with the reported conformations. However, it is worth to notice that an exhaustive benchmarking of DIMERO scores predicting DDIs was carried out in the work of Segura et al. (1) using atomic structures from the PDB (2).

The first analysis was performed on the VP16-Mediator-RNA polymerase II-TFII assembly in human (3)(EMDB code: EMD-5343). This structure (PDB code: 3J0K; see Figure S6A) is comprised by 12 distinct proteins and was used as an example of Scenario 1 (i.e., a hybrid model is proposed, and 3DIANA evaluates the likelihood of the proposed domain-domain interactions). From the proposed hybrid model, and in terms of physical binding between the protein domains, a total of 41 DDIs were observed. Out of these 41 domain pairs, 18 of them had a High Confidence Prediction level, and 23 had a Medium Confidence Prediction level. No proposed physical interaction was found to have a low likelihood. Therefore, this is the case of a proposed hybrid model that is very much in agreement with all previous interactomics knowledge as measured by DIMERO.

In the second example (Figure S7), the 'Domain-Domain Binding Analysis' tool was applied to the 40S-eIF1A-eIF1 complex from yeast (4) (EMDB code: EMD-3047). This structure (PDB code: 3JAM) is comprised by 36 different proteins and was also used as an example of Scenario 1. 3DIANA reported 49 interactions between the different protein domains where 46 of these domain pairs were classified as High Confidence Prediction (HCP), 2 of the binding domains as Medium Confidence Prediction and, finally, only one domain-domain interaction was classified as Not Significant (NS).

In these previous two examples we have shown how predictions of 3DIANA by means of DIMERO scores agrees with previously reported models. However, DIMERO is an statistical approach, and in some cases DIMERO scores may also lead to false predictions. To illustrate this case, we have analyzed the structure of the bacterial contractile nanotube present in *Pseudomonas aeruginosa* (5) (EMDB code: EMD-6270), showing the results in Figure S8. The atomic structure (PDB code: 3J9Q) is comprised of 48 chains represented by 2 unique proteins. The analysis found a total number of 234 interactions between the chains all of them involving a single DDI, thus the total number of DDI was also 234, but all of them were predicted as NS. The reason for this behavior is that only two different types of domain bindings were found: interactions between Phage_sheath_1 and Phage_tube domains and interactions between Phage_sheath_1 and Phage_sheath_1 domains. Both types of interactions are classified as NS using DIMERO and consequently all DDIs are classified as NS.

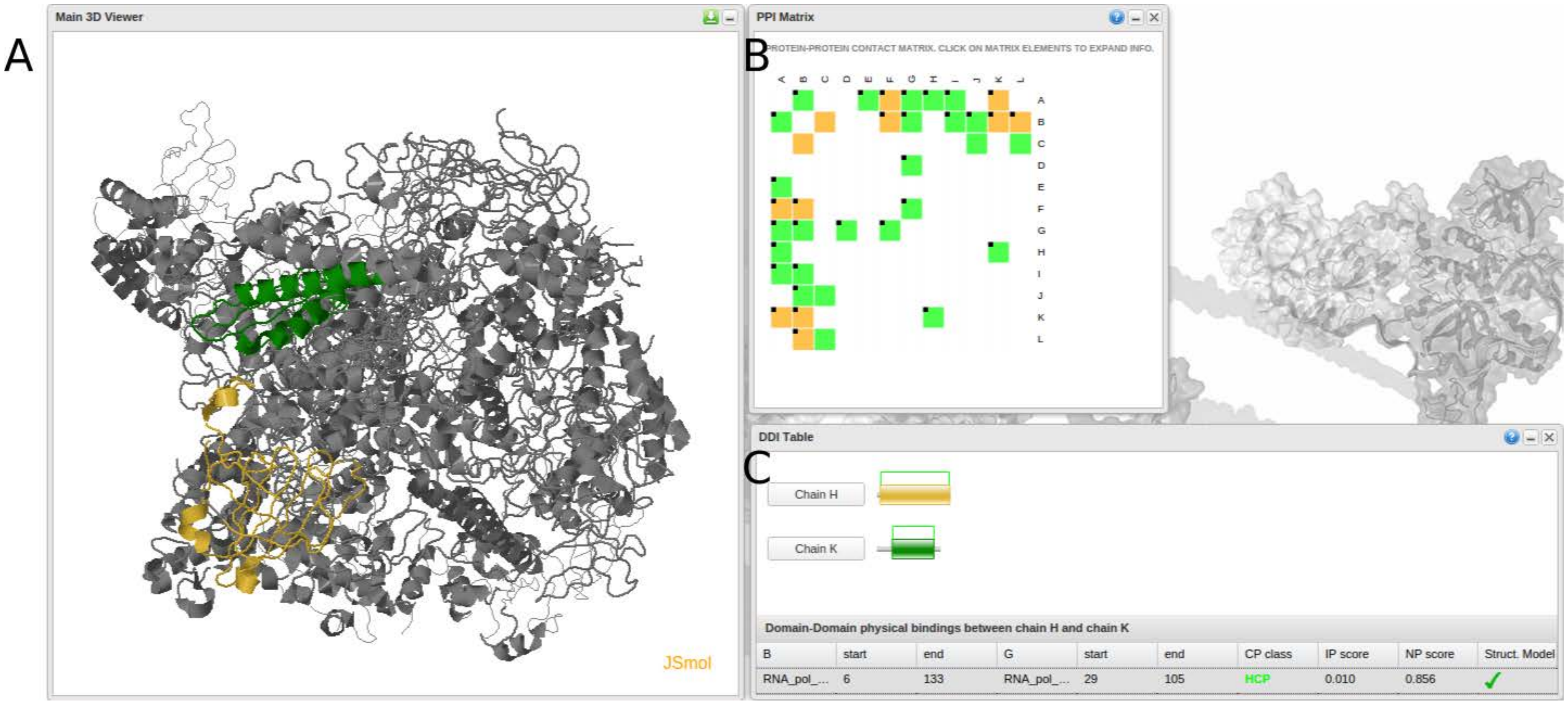

Figure S6: 3DIANA 'Domain-Domain Binding Analysis' results of the VP16-Mediator-RNA polymerase II-TFII assembly in human. (A) Atomic structure of the VP16-Mediator-RNA polymerase II-TFII complex (EMDB code: EMD-5343; PDB code: 3J0K). The 3D structure is displayed with the Jsmol viewer integrated in the 3DIANA web platform. (B) Protein-Protein Interaction Matrix displaying those interactions where at least a domain-domain binding occurs. The color code represents the best scored DDI using DIMERO approach (see Section 2.2 main manuscript) (C) Domain-Domain Interaction Table between chain H and chain K, only one DDI involving a RNA_pol_Rpb8 and RNA_pol_L_2 domain was observed between these chains. This domain pair was scored as HCP (see Section 2.2 main manuscript).

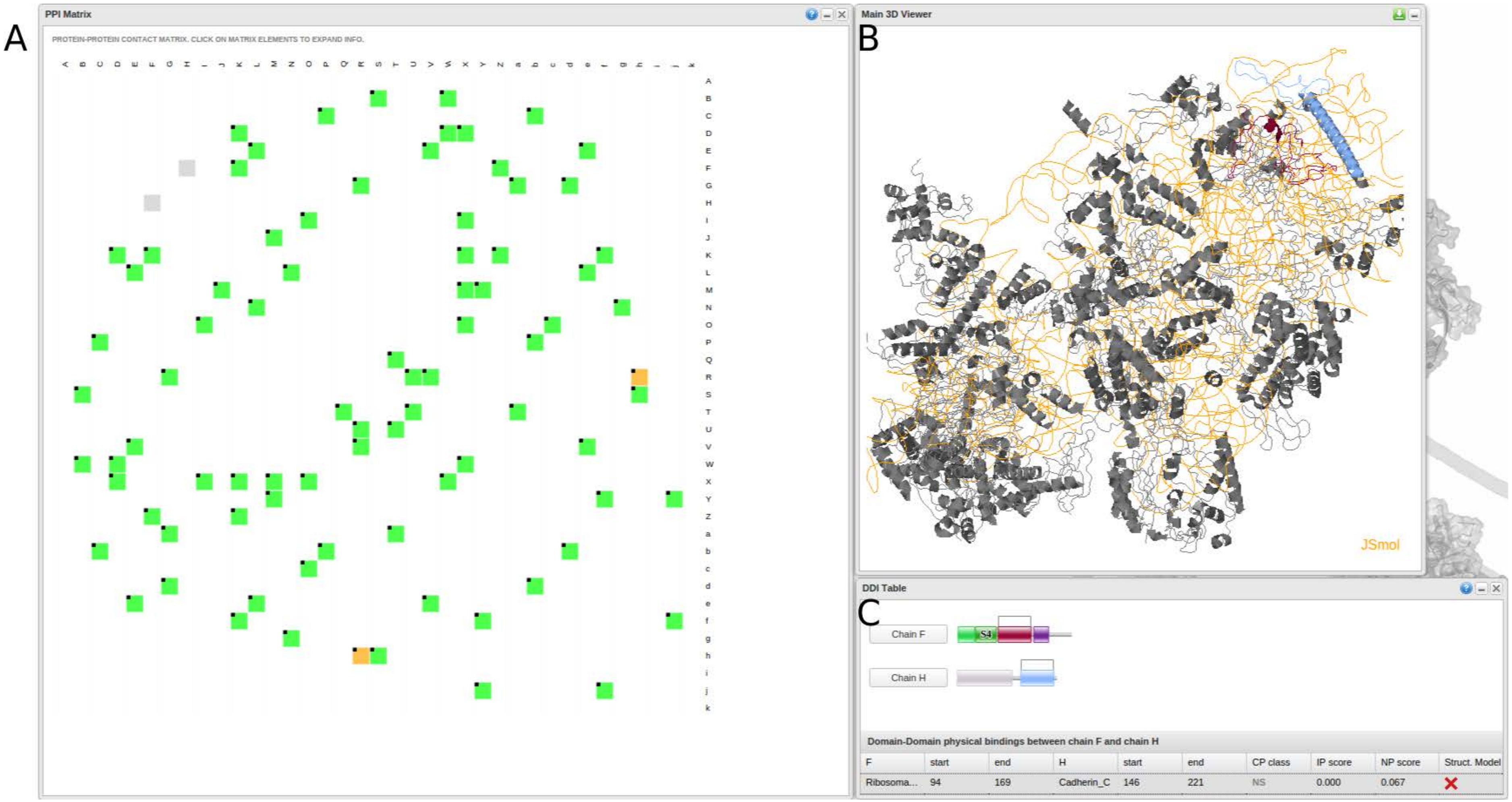

Figure S7: 3DIANA 'Domain-Domain Binding Analysis' results of the 40S-eIF1A-eIF1 complex in yeast. (A) Protein-Protein Interaction Matrix displaying those interactions where at least a domain-domain binding occurs. The color code represents the best scored DDI using DIMERO approach (see Section 2.2 main manuscript). (B) Atomic structure of the 40S-eIF1A-eIF1 complex (EMDB code: EMD-3047; PDB code: 3JAM). The 3D structure is displayed with the Jsmol viewer integrated in the 3DIANA web platform. (C) Domain-Domain Interaction Table between chain F and chain H, only one DDI involving a Ribosomal_S4e and Cadherin_C domain was observed between these chains. This domain pair was scored as NS (see Section 2.2 main manuscript).

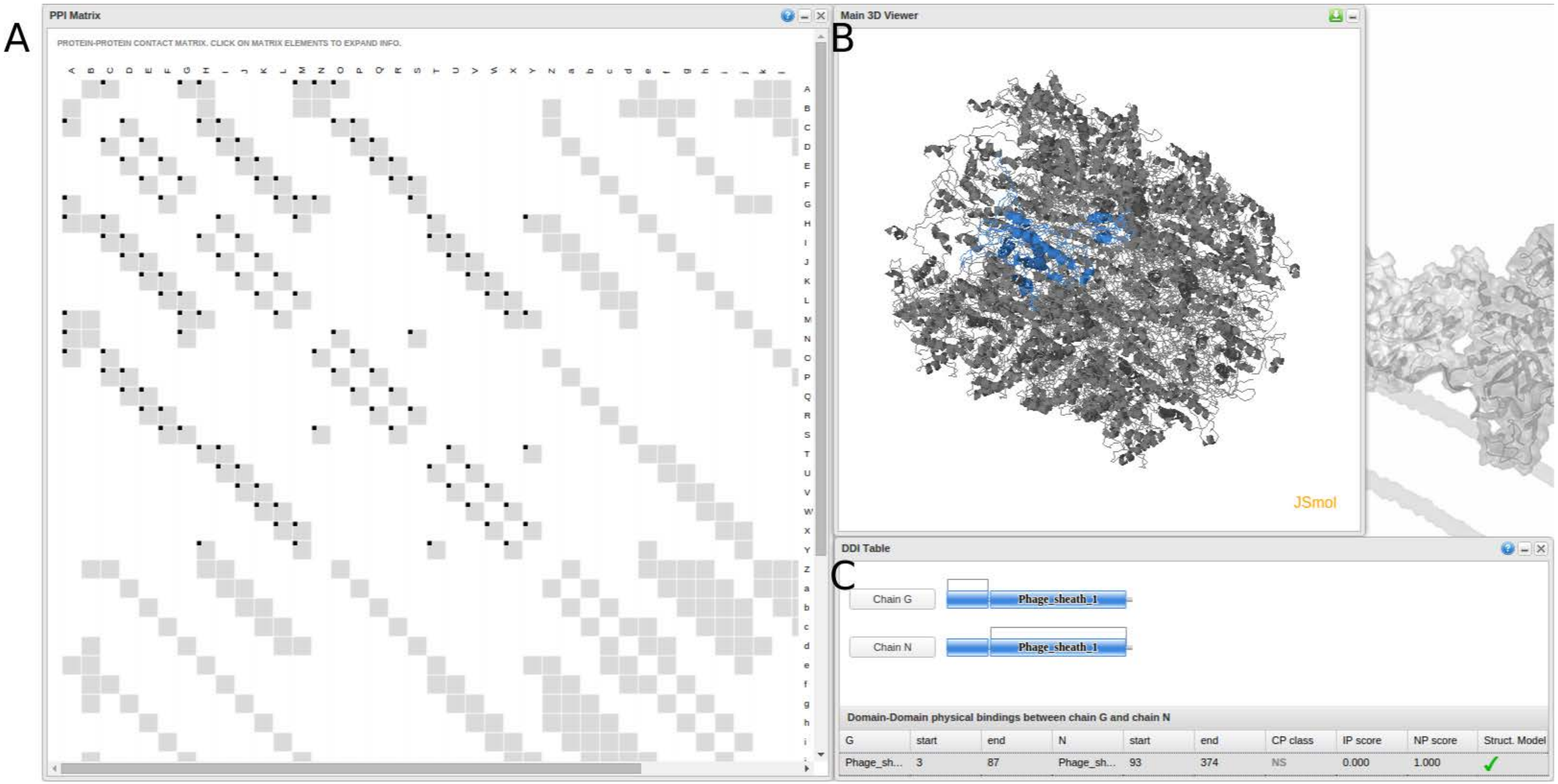

Figure S8: 3DIANA 'Domain-Domain Binding Analysis' results of bacterial contractile nanotube. (A) Protein-Protein Interaction Matrix displaying those interactions where at least a domain-domain binding is predicted to occur. The color code represents the best scored DDI using DIMERO (see Section 2.2 main manuscript). (B) Atomic structure of the bacterial contractile nanotube (EMDB code: EMD-6270; PDB code: 3J9Q). The 3D structure is displayed with the Jsmol viewer integrated in the 3DIANA web platform. (C) Domain-Domain Interaction Table between chain G and chain N, only one DDI involving two Phage_sheath_1 domains was observed between these chains. This domain pair was scored as NS (see Section 2.2 main manuscript).

References

1. Segura, J., C. O. Sorzano, J. Cuenca-Alba, P. Aloy, and J. M. Carazo. 2015. Using neighborhood cohesiveness to infer interactions between protein domains. Bioinformatics.
2. Gutmanas, A., Y. Alhroub, G. M. Battle, J. M. Berrisford, E. Bochet, M. J. Conroy, J. M. Dana, M. A. Fernandez Montecelo, G. van Ginkel, S. P. Gore, P. Haslam, R. Hatherley, P. M. Hendrickx, M. Hirshberg, I. Lagerstedt, S. Mir, A. Mukhopadhyay, T. J. Oldfield, A. Patwardhan, L. Rinaldi, G. Sahni, E. Sanz-Garcia, S. Sen, R. A. Slowley, S. Velankar, M. E. Wainwright, and G. J. Kleywegt. 2014. PDBe: Protein Data Bank in Europe. Nucleic acids research 42:D285-291.
3. Bernecky, C., P. Grob, C. C. Ebmeier, E. Nogales, and D. J. Taatjes. 2011. Molecular architecture of the human Mediator-RNA polymerase II-TFIIF assembly. PLoS Biol 9:e1000603.
4. Llacer, J. L., T. Hussain, L. Marler, C. E. Aitken, A. Thakur, J. R. Lorsch, A. G. Hinnebusch, and V. Ramakrishnan. 2015. Conformational Differences between Open and Closed States of the Eukaryotic Translation Initiation Complex. Mol Cell 59:399-412.
5. Lin, D. Y., J. Diao, and J. Chen. 2012. Crystal structures of two bacterial HECT-like E3 ligases in complex with a human E2 reveal atomic details of pathogen-host interactions. Proceedings of the National Academy of Sciences of the United States of America 109:1925-1930.
